# Isolation of bacterial extracellular vesicles from raw samples using a portable microstructured electrochemical device

**DOI:** 10.1007/s13346-025-01954-1

**Published:** 2025-08-26

**Authors:** Valeria Mantella, Siiri Bienz, Finn Brigger, Edouard Baulier, Marie Ramus, Nicole Zoratto, Steffen Honrath, Kumar Naresh, Sibilla Sander, Jörn Dengjel, Renato Zenobi, Vadim Krivitsky, Jean-Christophe Leroux

**Affiliations:** 1 Laboratory of Drug Formulation and Delivery, Institute of Pharmaceutical Sciences, Department of Chemistry and Applied Biosciences, ETH Zürich, Zürich, 8093 Switzerland; 2 Laboratory of Organic Chemistry, Institute of Pharmaceutical Sciences, Department of Chemistry and Applied Biosciences, ETH Zürich, Zürich, 8093 Switzerland; 3 OM Pharma SA, Meyrin, 1217 Geneva Switzerland; 4 Department of Biology, University of Fribourg, Chemin du Musée 10, Fribourg, 1700 Switzerland; 5 Acytronix GmbH, Wagistrasse 18, Schlieren, 8952 Switzerland

**Keywords:** Extracellular vesicles, Bacteria, Electrochemical device, Ultracentrifuge, Immune selectivity

## Abstract

Bacterial extracellular vesicles (EVs) are nanosized vesicles released by both Gram-negative and Gram-positive bacteria, playing critical roles in microbial communication, host-pathogen interactions, and immune modulation. Despite their significance in research and clinical applications, conventional isolation methods, such as ultracentrifugation (UC), are often slow, labor-intensive, and susceptible to contamination. In this study, we evaluated a novel portable microstructured electrochemical device (PMED) designed for rapid and selective bacterial EV isolation directly from biological samples. Using immunoaffinity-based capture and voltage-triggered release, the device-isolated EVs from Gram-negative *Escherichia coli* (*E. coli*), Gram-positive *Lactobacillus fermentum* (*Lb. fermentum*) culture supernatants and from urine samples spiked with *E. coli* , showing superior purity compared to UC. Characterization through nanoparticle tracking analysis (NTA), dynamic light scattering (DLS), and Western blot confirms enhanced selectivity and reduced contaminants. Functional assays demonstrated that device-isolated *Lb. fermentum* EVs selectively activated Toll-like receptor 4 (TLR4) without triggering TLR2, unlike UC-isolated EVs, suggesting a more refined immunomodulatory effect. These findings highlight the device’s translational potential for EV-based diagnostics, particularly for noninvasive urinary tract infection detection, and its broader applications in studying bacterial communication and immune regulation.

**Graphical abstract:**

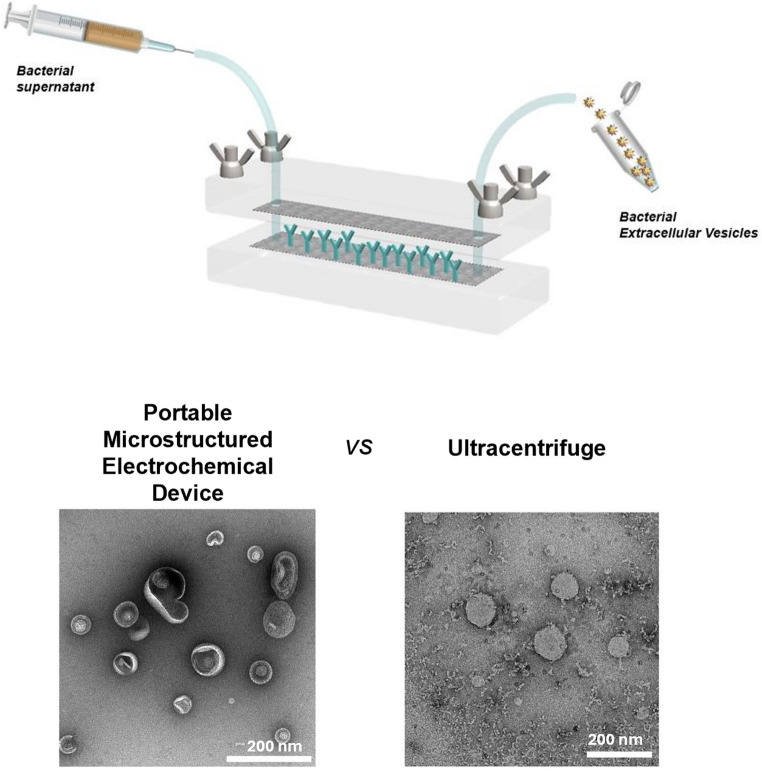

**Supplementary Information:**

The online version contains supplementary material available at 10.1007/s13346-025-01954-1.

## Introduction

Bacterial extracellular vesicles (EVs) are small, membrane-bound vesicles with diameters ranging from 20 to 250 nm [1, 2]. They are released by both Gram-negative (e.g. *Escherichia coli*, *E. coli*) and Gram-positive (*e.g. Lactobacilli*,* Lb.*) bacteria, through different mechanisms that vary according to the structural characteristics of the originating cell [1–3]. In the case of Gram-negative bacteria, EVs (also known as outer membrane vesicles, OMVs) primarily arise from the blebbing of the outer membrane [1–4]. This process is caused by several factors, including an imbalance in peptidoglycan biosynthesis, the intercalation of hydrophobic molecules, or explosive cell lysis induced by phage-derived endolysins that degrade the peptidoglycan cell wall [3, 4]. Given the thicker peptidoglycan layer in Gram-positive bacteria, it was initially believed that they could not release EVs [5]. However, recent research has elucidated that Gram-positive bacteria indeed produce EVs through budding from the cytoplasmic membrane [5]. Overall, bacterial EVs are able to enter host cells and deliver various biomolecules, including proteins, virulence factors, peptidoglycan, and nucleic acids [1, 2, 4–6].

Bacterial EVs can activate both the innate and adaptive immune systems, influencing a variety of biological functions including virulence, horizontal gene transfer, phage infection and transport of bacterial metabolites [1–7]. In host-microbe interactions, EVs contribute to immune modulation, epithelial cell adhesion, and the delivery of bacterial effectors that can influence host cell signaling and pathogen defense mechanisms [8, 9]. In particular, these EVs carry specific molecules known as pathogen-associated molecular patterns (PAMPs) which act as recognizable signals to the host immune system. Common PAMPs include lipoteichoic acid (LTA), lipopolysaccharides (LPS), lipopeptides, peptidoglycan, flagellin, and bacterial DNA [8, 10, 11]. Such molecules are recognized by highly conserved pattern recognition receptors, such as Toll-like receptors (TLRs). For instance, EVs derived from *Lactobacilli* have been shown to boost certain immune responses mediated by TLR2/1 and TLR4 receptors while dampening responses through TLR2/6 [10, 12].

Beyond their immunomodulatory roles, bacterial EVs have emerged as promising biomarkers for liquid biopsy applications, particularly for diagnosing conditions affecting the kidneys, bladder, and urogenital tract, including the prostate, uterus, and vagina [13–17]. While some pathogenic bacteria release EVs that contribute to infection and inflammation, these vesicles also serve as indicators of bacterial presence and activity within specific tissues [11].

Despite increasing interest in bacterial EVs and their diverse roles, their practical implementation remains constrained by the limitations of traditional isolation techniques [18, 19]. Generally, methods such as ultracentrifugation (UC), filtration, and size-exclusion chromatography are used to isolate these vesicles from their native biological samples [18, 19]. However, these approaches present several challenges: they are often time-consuming, retain impurities, offer limited yields, require chemical elution, as well as specialized laboratory equipment and expertise [18, 19]. Standard protocols for bacterial EV isolation typically start with ultrafiltration to remove bacterial debris, protein aggregates and other impurities. This is followed by UC steps to concentrate bacterial EVs and reduce the amount of non-bacterial EV-associated proteins, though with moderate success. In particular, isolated bacterial EVs may be contaminated with remaining bacterial flagella or large protein complexes, complicating downstream experiments and analyses [18, 19].

Building on these limitations, we have recently introduced a portable microstructured electrochemical device (PMED) for the immunoaffinity-based capture and voltage-triggered release of EVs [20]. In our previous work, this device was used to isolate different EV subpopulations from large volumes of various mammalian biofluids, as well as from the skin wounds of both healthy and diabetic mice [20]. Compared to UC, the method allowed to isolate EVs within a considerably shorter time, with enhanced purity and selectivity, while preserving their biological activity and structural properties [20].

In this study, we investigated the performance of the device for isolating EVs from bacteria cell culture supernatants and contaminated human urine. After isolation, the collected bacterial EVs were characterized for their physicochemical and biological properties. As a proof of concept, we used *E. coli* and *Lb. fermentum* cell culture supernatants for bacterial EV isolation. The selection was strategically made to demonstrate the versatility and robustness of the device across bacterial types with distinct EV biogenesis mechanisms, surface markers and biophysical properties. Both bacterial species are clinically significant. *E. coli* EVs serve as biomarkers for urinary tract infections (UTIs) and systemic infections [17, 21], while *Lb. fermentum* EVs have potential applications in probiotic therapies and modulation of inflammatory responses [22, 23].

## Materials and methods

### Materials

3-Aminopropyldimethylethoxysilane (S00750-5 g) was purchased from Fluorochem (UK). Mouse monoclonal anti-CD9 antibody (C-4) (sc-13118), mouse monoclonal anti-TSG101 antibody (sc-7964), were purchased from Santa Cruz Biotechnology (USA). Mouse monoclonal anti-bacterial outer membrane protein-A antibody (OMPA11-M), rabbit polyclonal anti-bacterial outer membrane protein-C antibody (BS-20213R), mouse monoclonal anti-bacterial *E. coli* elongation factor TU antibody (LS-C128699-100), rabbit polyclonal anti-bacterial flagellin antibody (316137), were purchased from Anawa CliniSciences Group (Switzerland). Rabbit polyclonal anti-bacterial surface layer protein antibody (AA 301–400) was purchased from Antibodies Online (USA). Polyclonal goat anti-mouse immunoglobulins/HRP secondary antibodies (P0447) were purchased from Dako (Denmark) and polyclonal goat anti-rabbit immunoglobulins/HRP secondary antibodies (ab97051) were purchased from abcam (CB2 0AX, United Kingdom). Phosphate buffered saline (PBS, KH_2_PO_4_ 1 mM, NaCl 155 mM, Na_2_HPO_4_.7H_2_O 3 mM, pH 7.4) was purchased from Thermo Fisher Scientific (USA). Silver/silver chloride paste (60/40), skim milk powder, bovine serum albumin (BSA), tris(hydroxymethyl)aminomethane (Tris) base, polysorbate 20, and ethanol absolute (> 99.8%), were purchased from Sigma–Aldrich (USA). SYLGARD 184 silicon elastomeric kit was purchased from Dow Corning (USA). De-ionized water (DIW) was supplied by a Merck Millipore MilliQ Direct-Q8 (USA). All the chemicals were used as received without further purification.

### Fabrication of the PMED

The PMED was fabricated as described previously [20]. Briefly, the device consists of a three-dimensional (3D) carbon electrode with a high surface area embedded in a poly(dimethylsiloxane) (PDMS)-based microchannel. The micro-carbon fiber (µCF) electrode is constructed from Freudenberg H23 carbon paper (22 cm × 30 cm, catalog number: 1590042, Freudenberg Performance Materials SE & Co. KG., Germany). The fluidic channel integrates silicon tubing to facilitate the flow of biosamples, which are injected using syringes. The initial step in the device assembly is the fabrication of the microchannels. Two ethanol-washed molds were used for this purpose. Double-sided adhesive carbon tape (8 mm × 20 m, cat. AGG3939, Agar Scientific Ltd, UK) was applied to the molds, and µCF electrodes (Freudenberg H23, 22 cm × 30 cm, 0.15 mm thickness, cat. 1590042, Freudenberg Performance Materials SE & Co. KG, Germany) were positioned on top of the tape. For the reference electrode, a thin 3–4 mm layer of Ag/AgCl paste (60/40% m/m, 901773-50G, Sigma-Aldrich) was applied and then dried in an oven at 80 °C. Silicon tubes (1 mm inner diameter, 3 mm outer diameter, 4.5 cm length, Millipore) were inserted into the counter electrode using needles (100 Sterican ⌀ 0.90 × 50 mm, 20 G×2″, Braun Injekt, Germany). PDMS (50 g) and a curing agent (5 g) were mixed, creating a whitish mixture that was poured onto the molds. The sides of the molds were delimited by microscope slides (26 × 76 × 1 mm, Menzel X50, Thermo Fisher Scientific) covered in aluminum foil. PDMS curing occurred at 80 °C for 1 h. The two semi-channels were then carefully removed, and the electrodes were thoroughly washed with ethanol and deionized water. The resistance of the electrodes and the quality of the contacts were measured using a Digital Multimeter (UNI-T UT61E, Unitrend Technology, China). The conductivity of the working electrode and counter electrode) was approximately 3 Ω cm^− 1^.

### Functionalization of the µCF electrodes

The functionalization of the µCF electrodes has been described in our previous publication [20]. The semi-channels with embedded µCF electrodes were cleaned with 40 mL of ethanol and dried in a vacuum oven at 100 °C for 30 min. A radio frequency (RF) source at 60 W for 1 min, at an oxygen gas pressure of 30 Pa (CY-P2L-B150, Zhengzhou CY Scientific Instrument Co., China), was then used to induce the formation of hydroxyl groups. Silanization was performed via gas-phase chemical adsorption of 300 µL 3-Aminopropyldimethylethoxysilane (APDMES) for 16 h at 100 °C and 0 Pa in a covered glass Petri dish, followed by heating to 105 °C under vacuum for 2 h. The semi-channels were subsequently washed with 50 mL of ethanol and dried under vacuum at 100 °C in a vacuum oven (KVTS11, Salvis, AG., Switzerland).

### PMED channel assembly

The assembly and characterization of the PMED channels were performed as described before [20]. The two semi-channels were aligned and combined to form a fluidic channel, then secured between two clear poly(methyl methacrylate) lids (105 mm × 55 mm × 5 mm) using six bolts and nuts. Steel needles were inserted into the PDMS to penetrate the µCF electrodes without touching the fluidic channel and tubing. The fluidic system was flushed with 5 mL of deionized water and 5 mL of PBS. The metal needles were connected to a potentiostat (EmStat3 Blue, PalmSens BV, Netherlands).

### Preparation of *E. coli* samples

The manufacture step involves the bacterial growth via successive unit operations from bacterial cell bank stored at -80 °C up to 400–600 L fermenters stage. The culture medium (200 mL) was collected at the end of the fermentation processes: optical density range 20.00–25.00, measured at 700 nm, (V-730, JASCO, Japan) with > 1.5 × 10^10^ cells/mL. The culture medium was spun at 10,000 x *g* for 20 min at a temperature of 4 °C (Allegra X-30R, Beckman Coulter, USA). The supernatant was then collected, filtered with 0.2 μm pore-size filter (567 − 0020 reference, Rapid Flow Nalgene, Thermo Fisher Scientific) and used for subsequent EVs isolation experiments. In addition, 1 mL of sterile supernatant was plated on Tryptone Soy Agar plate (43011 reference, BioMérieux, France) and incubated for 48 h at 37 °C (IF75, Memmert, Germany) to check absence of bacteria cells.

### Preparation of *Lb. fermentum* I3929 samples

Bacteria were grown via successive culture steps from bacterial cell bank stored at -80 °C up to 800-1000 mL lab scale fermenter. The culture medium (250 mL) was collected at the end of the fermentation processes (Multifors, INFORS HT, Switzerland) at optical density values of 3.00-3.50, measured at 700 nm, (NanoDropOne C, Thermo Fisher Scientific) with > 1.0 × 10^9^ cells/mL. The culture medium was spun at 6000 x *g* for 30 min at 10 °C (Allegra X-30R, Beckman Coulter). The supernatant was then collected, filtered with 0.2 μm pore size filters (Supor EKV membrane in mini Kleenpak capsule, KA02EKVP2S reference, PALL, USA) and used for subsequent EVs isolation experiments. The supernatant was also tested for sterility as mentioned above.

### General protocol for isolation by UC

For both bacterial strains, 25 mL of supernatant were centrifuged at 110,000 x *g* and 4 °C for 60 min using an Optima XE-90 ultracentrifuge (Beckman Coulter), equipped with a Type 45 Ti Fixed-Angle Titanium Rotor. The resulting pellet was resuspended in 25 mL PBS, pooled and subjected to a second UC run under the same conditions. The final pellet was resuspended in 1 mL of PBS and stored at 4 °C. After characterization, the aliquots were stored at -20 °C.

#### Isolation of bacterial EVs from *E. coli* and *Lb. fermentum* I3929 samples with the PMED

For the isolation of EVs from *E. coli* and *Lb. fermentum* I3929 with the device, the electrodes were coated with 20 µg of the antibody, (100 µg/mL in 200 µL PBS), using a 1-mL syringe. An empty 1-mL syringe was used at the other end of the channel to collect the solution. The antibody solution was incubated for 1 h at room temperature and transferred between the two syringes every 10 min, facilitating mixing and re-distribution of the antibodies. This dynamic flow approach helps ensuring even deposition of the antibodies on the carbon surface.

After incubation, the non-adsorbed antibody solution was removed using a 1 mL syringe, followed by washing with 5 mL of PBS to remove remaining non-bound antibodies. Then, 25 mL of *E. coli* supernatant or of *Lb. fermentum* supernatant were injected into the fluidic channel at a rate of 0.3- 1 mL/s and recirculated ten times. The remaining solution was then withdrawn, and 40 mL of PBS were injected at 0.3 mL/s to remove contaminants. For the release step, 1 mL of PBS was injected and then withdrawn from the device. Before the electrochemical release, a 1-mL syringe filled with PBS was connected to one end of the device, and an empty 1 mL-syringe was connected to the other end. The device’s needles were connected to a potentiostat, and a potential of − 1.5 V was applied to the working electrode for 45 s while the solution was cycled back and forth through the channel for 10 times. This process was repeated with the electrical poles reversed. As a negative control, only PBS solution (no bacteria or additives) was processed using the PMED device, and no particles were detected by NTA. The same experimental conditions described for the experiment with *E. coli* supernatant, were applied.

#### Preparation of *E. coli*-contaminated urine sample and isolation of bacterial EVs with the PMED

Urine (Human Urine Male, BioIVT, LOT# NMN563177, UK) biosample was stored at − 80 °C. The initial concentration of *E. coli* culture media was 10^7^ CFU/mL. Given that counts as low as 10^2^ CFU/mL in urine can indicate an infection [24], a series of 1:10 serial dilutions was performed to progressively reduce the bacterial concentration. First, 1 mL of the 10^7^ CFU/mL culture was transferred into 9 mL of sterile PBS. The mixture was then thoroughly mixed. From this diluted sample, 1 mL was transferred into another tube containing 9 mL of PBS. This process was repeated until the bacterial concentration reached 10^4^ CFU/mL. To achieve a final concentration of 10^2^ CFU/mL in 25 mL of urine, 0.25 mL of the 10^4^ CFU/mL dilution was added directly to the urine sample and mixed thoroughly. Finally, the resulting 25 mL of contaminated urine sample were introduced into the device channels without any pre-purification step, and EVs were isolated, following the protocol described in Section “Isolation of bacterial EVs from *E. coli* and *Lb. fermentum* I3929 samples with the PMED”. To exclude the presence of EVs from Gram-negative bacteria and eukaryotic cells, three devices were modified with anti-OmpA and tested with urine only. Following the application of standard electrical potential, no particles were detected by NTA.

### Atomic force microscopy (AFM)

AFM topography imaging was conducted in tapping mode under ambient conditions using an AFM system from Horiba, France. Si cantilevers from AppNano (USA) with a spring constant of 2.7–44 N/m and a resonance frequency of 68–290 kHz were employed. Imaging was performed at a scan rate of 0.5–5 Hz. AFM topography images were analyzed using the open-source software Gwyddion (Version 2.49, Czech Republic) [25]. The root mean square (RMS) was calculated based on Eq. 1:

1 $$\:RMS=\:\sqrt{\frac{1}{N}\sum\:_{j=1}^{N}{r}_{j}^{2}}$$

Where:

- *N* = number of data points.
- *j* = index.
- $$\:{r}_{j}=\:{z}_{j}-\:\stackrel{-}{z}$$, where:
  - $\:{z}_{j}=$ *height at a specific point j*.

$\:\stackrel{-}{z}=\:$*mean height of the surface* [25].

### Preparation of the antibody, vesicles and antibody-vesicle complex solution on mica substrate for AFM imaging

The mica surface underwent the same functionalization treatment as the carbon fibers, which included oxygen plasma and silanization [20]. To prepare the OmpA antibody solution, 20 µL of the antibody solution were drop-cast directly from the original batch onto the mica surface and then dried at 40 °C for 48 h. For the *E. coli* EVs, 25 mL of the original supernatant were processed through UC (Section “General protocol for isolation by UC”). The resulting pellet was resuspended in 1 mL of PBS. Subsequently, 20 µL of this EV solution were drop-cast onto the mica surface and dried at 40 °C overnight. The antibody-vesicle complexes were prepared by gently mixing post-UC EVs with OmpA antibodies from their original stock solution at a volume ratio of 1:2 (EVs:OmpA), resulting in a final volume of 150 µL. The mixture was incubated at room temperature for 30 min to ensure efficient binding of the antibodies to the OmpA molecules on the EV surfaces. Following incubation, 20 µL of resulting solution were then drop-cast directly onto the mica surface and dried at 40 °C overnight.

### Preparation of the antibody-vesicle complexes on device fibers for AFM imaging

To prepare the OmpA antibody-EV complex on the device fibers, the isolation procedure described in Section “Isolation of bacterial EVs from *E. coli* and *Lb. fermentum* I3929 samples with the PMED” was followed without applying electrical voltage, as the objective was to image vesicle binding to the antibodies rather than their release. After introducing 25 mL of supernatant into the antibody-modified channels and performing a PBS wash, the fibers—now containing the antibody-vesicle complexes—were carefully removed from the carbon tape using tweezers, separated, and placed on a glass substrate. The substrate was then incubated at 40 °C overnight, covered with a cup to prevent dust contamination. Once dried, the fibers were transferred onto an atomically flat mica surface and placed under the AFM microscope for imaging.

### Nanoparticle tracking analysis (NTA)

NTA was used to estimate the particle concentration and the size profile of the isolated bacterial EVs. For the measurement, a ZetaView^®^ PMX 120-Z instrument (Particle Metrix GmbH, Germany), featuring a 405-nm laser source and a CMOS camera, was used. The NTA device was calibrated with a 1:500,000 dilution of silica beads. To measure our samples, the shutter was set to 150, sensitivity to 85, and the frame rate to 30. Data were analyzed with the ZetaView software version 8.04.04 SP2, (Particle Metrix GmbH), applying a bin class width of 5 nm, a minimum brightness of 25, a minimum area of 5, and a maximum area of 1000, A 1:50 (v/v) dilution of the EV samples was inserted using a syringe and measured for each sample in triplicates. The Savitzky-Golay filter (Points of Window 10) was used for smoothing data by fitting successive subsets of adjacent data points with a second-degree polynomial function. The NTA plots show the mean particle size and concentration, derived from 3 independent experiments, conducted each in triplicates.

### Dynamic light scattering (DLS)

The hydrodynamic diameter (nm) and zeta-potential (mV) of the vesicles were measured on a Malvern Zetasizer Advance Pro 722 instrument (Malvern Panalytical, UK) operating at a scattering angle of 179° and at a temperature of 25 °C. The hydrodynamic diameters were determined based on light scattering intensity, with samples diluted 1:10 (v/v) PBS in nanopure water, and measured in 70 µL micro UV-cuvettes (Brand GmbH + CO. KG., Germany). The zeta-potentials were performed *via* laser Doppler anemometry, with samples diluted with 10% (v/v) PBS in nanopure water and analyzed in 800 µL Folded Capillary Zeta Cell cuvettes (Malvern Panalytical GmbH, Germany).

### Transmission electron microscopy (TEM)

TEM was used to image EVs. To this end, 300-mesh copper grids with ultrathin carbon support film were negatively glow-discharged in a Pelco EasiGlow discharge system (Ted Pella Inc., USA) at 25 mA for 30 s at a pressure of 38 Pa. Subsequently, 4 µL of the EV solution were deposited onto the grids for a 30 s absorption period. The grids were treated with a uranyl acetate staining solution and washed with distilled water. Excess liquid was carefully removed using filter paper, and the grids were dried under ambient conditions. The samples were imaged with a Tecnai F20 field emission gun microscope equipped with a combination of a CCD camera (ORIUS SC200 2 K, Gatan, USA) and a direct electron detector (Falcon II 4 K, Thermo Fisher Scientific) at an acceleration voltage of 120 kV. The micrographs were acquired in the bright field mode.

### Cryo transmission electron microscopy (Cryo-TEM)

To prepare samples for cryo-TEM, lacey carbon EM grids, (Au 300 mesh, 150 μm, Byolist Scientific, USA), were glow-discharged in Pelco EasiGlow system (Ted Pella Inc) at 25 mA for 30 s at a pressure of 38 Pa. Subsequently, 3.4 µL of the EV solution were applied onto the carbon side of EM grid, which was then blotted for 2.0 s and plunge-frozen into the precooled liquid ethane: propane mixture with Vitrobot Mark IV (Thermo Fisher Scientific). This procedure applied a thin layer of amorphous ice to the samples, preserving them in their native state and shielding them from potential radiation damage. In order to obtain a good ice thickness, the blotting time was set to 2.5 s, the blotting force to 0, and the temperature to 22 °C. The cryo-TEM images were acquired at Titan Krios FEG (Thermo Fisher Scientific) at 300 kV using a direct electron detector (Falcon III 4k × 4k) (Thermo Fisher Scientific), which works in tandem with a Ceta 16 M 4k × 4k CMOS detector (Thermo Fisher Scientific) and K2 (Gatan, USA) with the Quantum LS energy filter (Gatan).

### Determination of bacterial EV protein concentration

The total protein content of bacterial EV samples was measured with the Micro BCA Protein Assay Kit (Thermo Fisher Scientific), according to the manufacturer’s instructions. The preparation of BSA standards is described in Table S1. Bacterial EVs samples were prepared by diluting 5 µL of sample with nanopore water to a volume of 150 µL. After addition of 150 µL Micro BCA reagent (25:24:1 (v/v), reagent A:B:C) per well, the plate was incubated at 37 °C for 90 min in the dark. The absorbance was measured at 562 nm using the Tecan Infinite M200 plate reader (Tecan, Switzerland). Protein concentration was determined by accounting for the dilution of the EV sample.

### Western blotting

Sample buffer (0.25 M Tris, 10% w/v SDS, 30% v/v glycerol, 0.02% w/v bromophenol blue, 5% v/v β-mercaptoethanol) was added 5:1 (v/v) to the bacterial EV samples to denature proteins and promote a sufficient flow on the gel. The samples were then heated for 5 min at 95 °C. Following this, the samples and the size marker were loaded on a 12% SDS polyacrylamide gel (total final volume loaded in each well was 15 µL). The protein samples (1–7 µg) were resolved at 80 V for approximately 1.5 h. The transfer to polyvinylidene fluoride membranes was performed with a Trans-Blot^®^ Turbo™ system at 25 V for 10 min (Bio-Rad, USA). Blocking was conducted for 2 h using Tris-buffered saline (20 mM Tris, 150 mM NaCl) with 0.1% (v/v) polysorbate 20 (TBS-T) and 5% (w/v) skim milk powder for OmpA, OmpC, flagellin, TSG101 and CD9. Instead, blocking was conducted for 2 h using 8% (w/v) BSA in tris-buffered saline (TBST) for Elongation TU, while 15% (w/v) BSA in TBS-T was used for ENO-1 and S-layer. These conditions were optimized to minimize non-specific antibody interactions while maintaining effective target binding. Following this incubation time, the blocking solution was discarded, and the membranes were incubated with primary mouse monoclonal anti-bacterial antibodies including OmpA and Elongation TU, or with primary rabbit polyclonal anti-bacterial antibodies including OmpC, flagellin, S-layer and ENO1 in TBS-T (1:200 v/v). Primary antibodies were incubated overnight at 4 °C. After three washes with TBS-T (20 min, room temperature), secondary horseradish peroxidase (HRP)-conjugated antibodies in TBS-T were added for 4 h at 4 °C. Antibody concentrations (v/v) are provided in Table S2. Membranes were incubated with Western Blotting Luminol reagent (Santa Cruz Biotechnology, USA) according to the manufacturer’s protocol and imaged using a ChemiDoc MP Imaging System (Bio-Rad).

### Nano flow cytometry (NanoFCM)

Bacterial EVs were labeled with 5 µM CellTrace™ Far Red dye (Thermo Fisher Scientific) in PBS, following a previously described protocol [26]. Briefly, samples were incubated overnight at 4 °C with the dye, followed by 15 min at 37 °C to promote acetate hydrolysis of the dye. Excess dye was removed by dialysis against PBS overnight at 4 °C using the Pur-A-Lyzer™ Mini 6000 Dialysis Kit (Sigma-Aldrich).

Samples were analyzed using a Flow NanoAnalyzer N30 instrument (NanoFCM Co., Ltd., UK). Light scattering and fluorescence from individual particles were collected for 1 min across three channels: 488/10, 525/40, and 670/30 using single-photon counting avalanche photodiode (SPCM APD) detectors. Channel alignment was performed with fluorescent 250 nm silica QC beads and particle size was determined according to standard operating procedures by generating calibration curves using the S16M-Exo silica nanosphere cocktail (both NanoFCM Co., Ltd.), which includes four distinct silica particle populations with diameters of 68 nm, 91 nm, 113 nm and 155 nm. Samples were measured with lasers set to 10/50 mW (488 nm) and 20/100 mW (638 nm) with a 10% side scatter decay. The sampling pressure was maintained at 1.0 kPa throughout the measurement. Before measurement, the samples were diluted in PBS to ensure a final event count ranging from 500 to 3200. The data was processed using the NanoFCM Professional Suite (version 2.3, NanoFCM Co., Ltd.) and the FlowJo software (version 10.9.0, BD Biosciences, USA). Experimental data are shown as a result of 3 independent experiments, conducted each in triplicates.

### Proteomic analysis—EVs proteome analysis

Two *Lb fermentum* EV samples (post-device) were dissolved in 1% (w/v) sodium deoxycholate in 50 mM ammonium bicarbonate buffer (pH 8), reduced with 1 mM dithiothreitol, and alkylated using 5.5 mM iodoacetamide for 10 min at room temperature. Proteins were in-solution digested with trypsin (Promega, USA). Sodium deoxycholate was precipitated using 50% TFA (trifluoroacetic acid), and tryptic peptides were purified by STAGE tips prior to LC–MS/MS measurements. These were performed on a QExactive HF-X mass spectrometer (Thermo Fisher Scientific) coupled to an EasyLC 1000 nanoflow-HPLC (Thermo Fisher Scientific). Peptides were separated on a fused silica HPLC-column tip (I.D. 75 μm, New Objective, self-packed with ReproSil-Pur 120 C18-AQ, 1.9 μm (Dr. Maisch, Germany) to a length of 20 cm), using a gradient of A (0.1% v/v formic acid in water) and B (0.1% v/v formic acid in 80% v/v acetonitrile in water). The mass spectrometer was operated in data-independent mode. After each survey scan (mass range m/z = 350–1200; resolution: 120,000), 28 DIA scans with an isolation width of 31.4 m/z were performed, covering a precursor range of 350 to 1200 m/z. AGC target value was set to 3 × 10^6^, resolution to 30,000, and normalized collision energy to 27%. Data were analyzed using Spectronaut software version 15.7 (Biognosys, Switzerland) with standard settings (without imputation) in direct DIA mode, using the reference proteomes uniprotkb_*Limosilactobacillus fermentum* and uniprotkb_Rabbit (UniProt, full length) and common contaminants.

### Cell stimulation assays

HEK-Blue™ hTLR2: hkb-htlr2 and HEK-Blue™ hTLR4: hkb-htlr4 (Invivogen, USA) were plated at a density of 5.10^4^ cells / well of a 96 well plate in 100 µL of culture medium (DMEM high glucose glutamax, Gibco, 61965-026 + 10% v/v fetal calf serum, Gibco A52567, 10% fetal bovine serum (FBS), Thermo Fisher Scientific). After 24 h of incubation, the medium was replaced with fresh medium, and various dilutions of EVs isolated from *Lb. fermentum* culture supernatant were added to the cells. After 24 h of incubation 37 °C under 5% CO_2_ atmosphere, SEAP activity was revealed using QUANTI-Blue reagent (Invivogen) and a Fluorescence plate reader (SpectraMax M3. Molecular Devices, USA) according to manufacturer’s instruction. Results were expressed by comparison between the basal Optical Density (OD) measured in cells supernatant without any treatment and OD obtained after cells stimulation. The NF-kB activity was calculated using Eq. 2

2 $$\:NF-\kappa\:B\:activity=\frac{\mathrm{O}\mathrm{D}\:\mathrm{o}\mathrm{f}\:\mathrm{s}\mathrm{t}\mathrm{i}\mathrm{m}\mathrm{u}\mathrm{l}\mathrm{a}\mathrm{t}\mathrm{e}\mathrm{d}\:\mathrm{c}\mathrm{e}\mathrm{l}\mathrm{l}\mathrm{s}}{\mathrm{O}\mathrm{D}\:\mathrm{o}\mathrm{f}\:\mathrm{c}\mathrm{e}\mathrm{l}\mathrm{l}\:\mathrm{c}\mathrm{u}\mathrm{l}\mathrm{t}\mathrm{u}\mathrm{r}\mathrm{e}\mathrm{d}\:\mathrm{w}\mathrm{i}\mathrm{t}\mathrm{h}\:\mathrm{m}\mathrm{e}\mathrm{d}\mathrm{i}\mathrm{u}\mathrm{m}}$$

This ratio was expressed as activity “relative to unstimulated cells”; a ratio equal to 1 corresponding to absence of NF-kB activity.

### Statistical analysis

The statistical analysis was conducted using the GraphPad Prism software version 8.0.0, (GraphPad Software, Inc., USA). The data were presented as mean ± SD. p-values were calculated using an unpaired t-test with a two-tailed distribution and unequal variance. *p < 0.05, **p < 0.01, ***p < 0.001, ****p < 0.0001.

## Results and discussion

### AFM imaging of the antibody-vesicle interaction

**Fig. 1:**
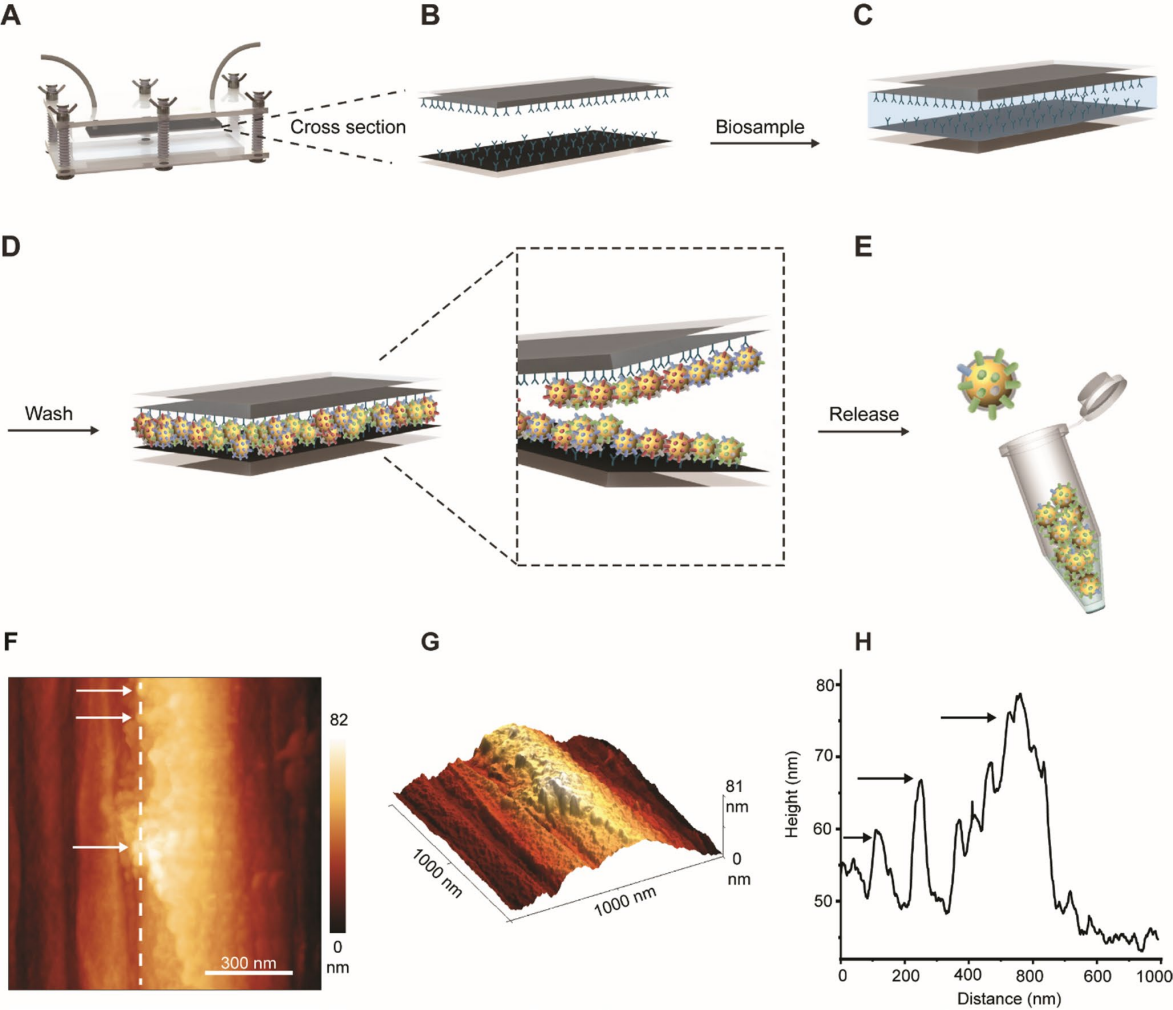
(**A**-**E**) Schematic representation of the PMED and operation process. (**A**) PMED setup, (**B**) antibody conjugation of the electrodes, (**C**) injection of the sample, (**D**) washing step and (**E**) controlled release of the attached EVs through the application of voltage. (**F**) AFM topography image of a carbon fiber incubated with *E. coli*-derived EV- anti-OmpA antibody complexes attached on top (after step D of the process). Area:1 µm^2^. Resolution: 516 × 516 pixels. (**G**) 3D representation of the AFM topography image shown in Panel F), displaying the presence of *E. coli*-derived EV- anti-OmpA antibody complexes on the carbon fiber surface. (**H**) Height profile of the carbon fiber surface along the line marked in Panel F). The arrows in Panels F) and H) indicate the location of the *E. coli*-derived EV- anti-OmpA antibody complexes on the carbon fiber

The PMED operates via a dual mechanism of EV capture through immunoaffinity and subsequent release triggered by electrical voltage (Fig. 1A-E). As previously described, the system comprises a fluidic channel featuring antibody-coated microstructured carbon electrodes (Fig. 1A-B) [20]. During operation, the biological sample flows through the channel (Fig. 1C), enabling selective capture of EVs by immunoaffinity. This process isolates EVs from the rest of the sample components, which are subsequently removed by washing with PBS (Fig. 1D). Finally, the captured EVs are released into PBS using a -1.5 V electrical voltage (Fig. 1E), and stored at 4 °C for a short period prior to downstream characterization. Three main processes contribute to the release of the EVs from the antibodies: (I) Electrical repulsion between the negatively charged EVs and the negative working electrode (WE). (II) Below − 1.23 V, water molecules are subjected to electrolysis, which produces H_2_ (g) and O_2_ (g) on the surface of the WE and the counter electrode (CE), respectively. These gases repel the EVs and prevent re-binding. (III) Local pH changes on the surface of the electrodes lead to conformational changes of the antibodies and weaken their affinity towards the EVs [20].

To isolate bacterial EVs from *E. coli* culture media, the device’s carbon electrodes were coated with an anti-OmpA antibody. OmpA is a porin, which is abundant in the outer membrane of many Gram-negative bacteria, including *E. coli*. It facilitates the passive diffusion of small molecules, like amino acids, across the membrane and plays a crucial role in maintaining membrane integrity [27, 28].

AFM was used to confirm and visualize the specific binding interactions between the captured *E. coli* vesicles and anti-OmpA antibodies on the carbon fiber (Fig. 1F-H). To allow vesicle-antibody binding, the *E. coli* culture medium was introduced into the device, where the carbon electrodes were coated with anti-OmpA antibodies (Section “Preparation of the antibody-vesicle complexes on device fibers for AFM imaging”). After passing the *E. coli* culture medium through the channels, the carbon fibers were carefully removed from the carbon tape. They were then dried at 40 °C on a clean glass substrate overnight before being transferred to an atomically flat mica surface for analysis. The coated fibers were compared with 5 control samples: mica substrate, pristine carbon fibers, OmpA antibodies on the mica substrate, EVs isolated via UC on the mica substrate, OmpA antibody-EV complexes on the mica substrate (Fig. S1 and Section “Preparation of the antibody, vesicles and antibody-vesicle complex solution on mica substrate for AFM imaging”). AFM imaging of individual antibodies on carbon fibers was not feasible due to the substantial intrinsic roughness of the fiber surface.

As shown in Fig. S1A-N, similar height was observed for the antibody-EV complexes on the carbon fibers and those on the mica substrate, suggesting successful imaging of the complexes on the fibers. In particular, the AFM topography image (Fig. 1F) and its corresponding 3D representation (Fig. 1G) revealed several globular features on the surface of the coated carbon fibers. The height profile along the fiber, presented in Fig. 1H, indicates that the globular features attain a height of up to 30 nm, consistent with the maximum height of the EV-protein complexes observed on the mica substrate (Fig. S1G and H). In contrast, the AFM topography image of a pristine carbon fiber (without antibodies and EVs) showed no globular features (Fig. S1I). The RMS roughness of the topography image of the carbon fiber incubated with the antibody-EV complexes (Fig. 1F) was 17.7 nm, whereas the uncoated fiber exhibited an RMS roughness of 8.4 nm (Fig. S1J). Additional AFM measurements of the carbon fibers incubated with the antibody–EV complexes revealed RMS roughness of 12.1 nm and 14.0 nm (Fig. S1K and S1M). These results confirm successful capture of the *E. coli*-derived EVprotein complexes by the carbon fibers.

### Isolation of bacterial EVs from Gram-negative *E. coli* and Gram-positive *Lb. fermentum*

As described above, the electrode surfaces were coated with anti-OmpA antibodies to isolate bacterial EVs from the Gram-negative *E. coli* culture medium. For *Lb. fermentum*, an anti-S-layer antibody was used to capture the corresponding bacterial EVs, taking advantage of its presence on the outer membrane of *Lactobacillus* species. S-layer glycoproteins self-assemble into a uniformly spaced pattern on the bacterial cell surface, where they play a key role in adhesion to intestinal epithelial cells and the mammalian extracellular matrix [29].

**Fig. 2:**
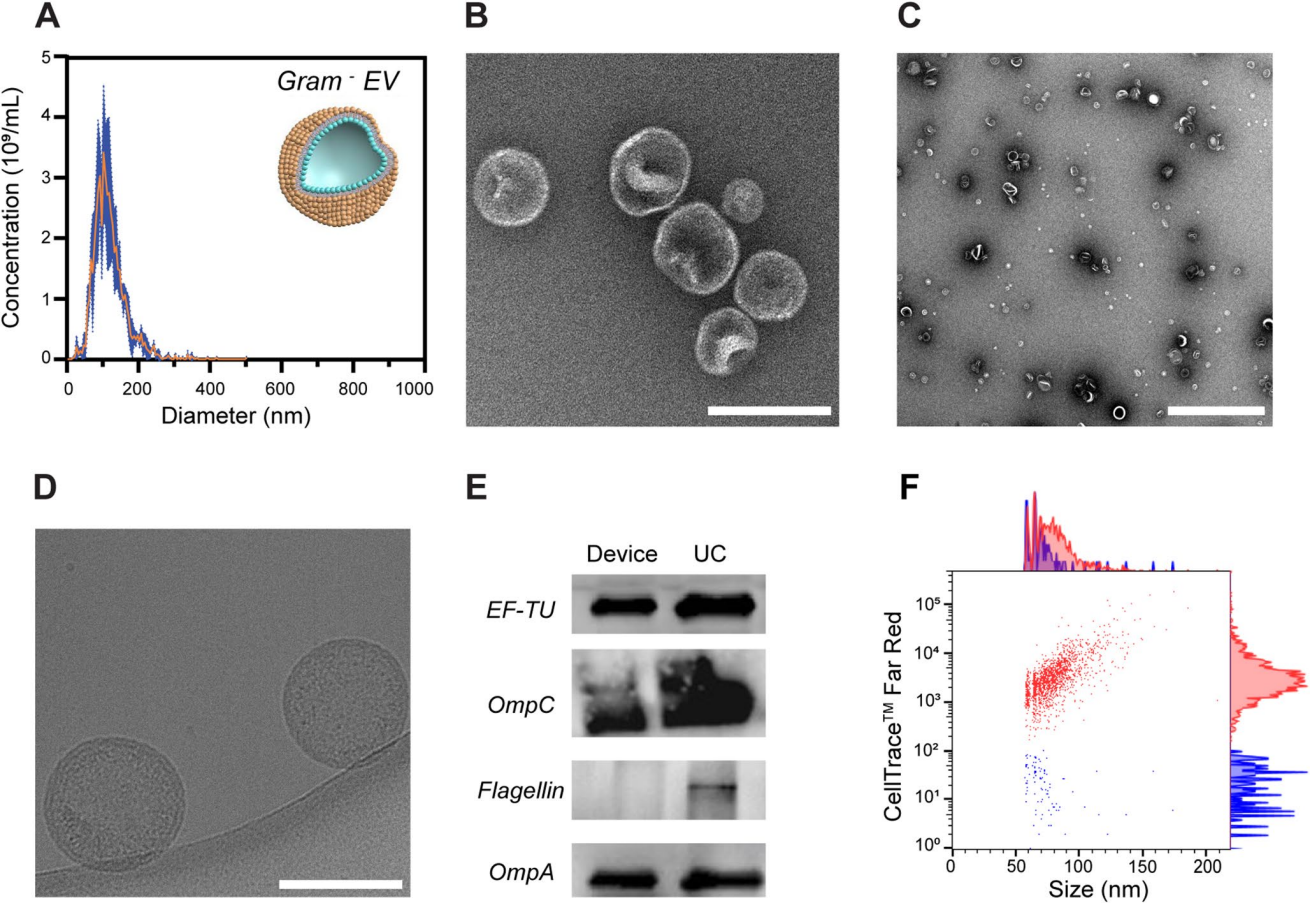
Characterization of bacterial EVs from Gram-negative *E. coli* supernatant. (**A**) NTA plot of EVs isolated using the device with an anti-OmpA antibody coating. Experimental data are expressed as mean (orange) ± SD (dark blue) (n = 3) Inset: schematic representation of a Gram-negative EV with the outer membrane colored in gold. (**B**) Representative high magnification TEM image of a field of isolated EVs in PBS. Scale bar: 200 nm. (**C**) Representative low magnification TEM image of a field of isolated EVs in PBS. Scale bar: 500 nm. (**D**) Representative high magnification cryo-TEM image of two isolated EVs in PBS. Scale bar: 200 nm. (**E**) Western blot analysis of bacterial EV protein markers (EF-TU, OmpC, flagellin and OmpA), showing the EV proteins isolated with the device in the left lane, and those isolated by UC in the right lane. (**F**) NanoFCM analysis of CTFR stained *E. coli*-derived EVs. A representative bivariate dot plot of size (nm, x axis) vs CTFR (y axis) shown, with corresponding histograms displayed alongside. CTFR-positive events are shown in red, while CTFR-negative events are shown in blue. A representative plot is reported, with the mean values of 91.9 ± 2.8% for CTFR-positive events and 8.1 ± 2.8% for CTFR-negative events. Original uncropped Western blots are provided in the supporting information (Fig. S6)

**Fig. 3:**
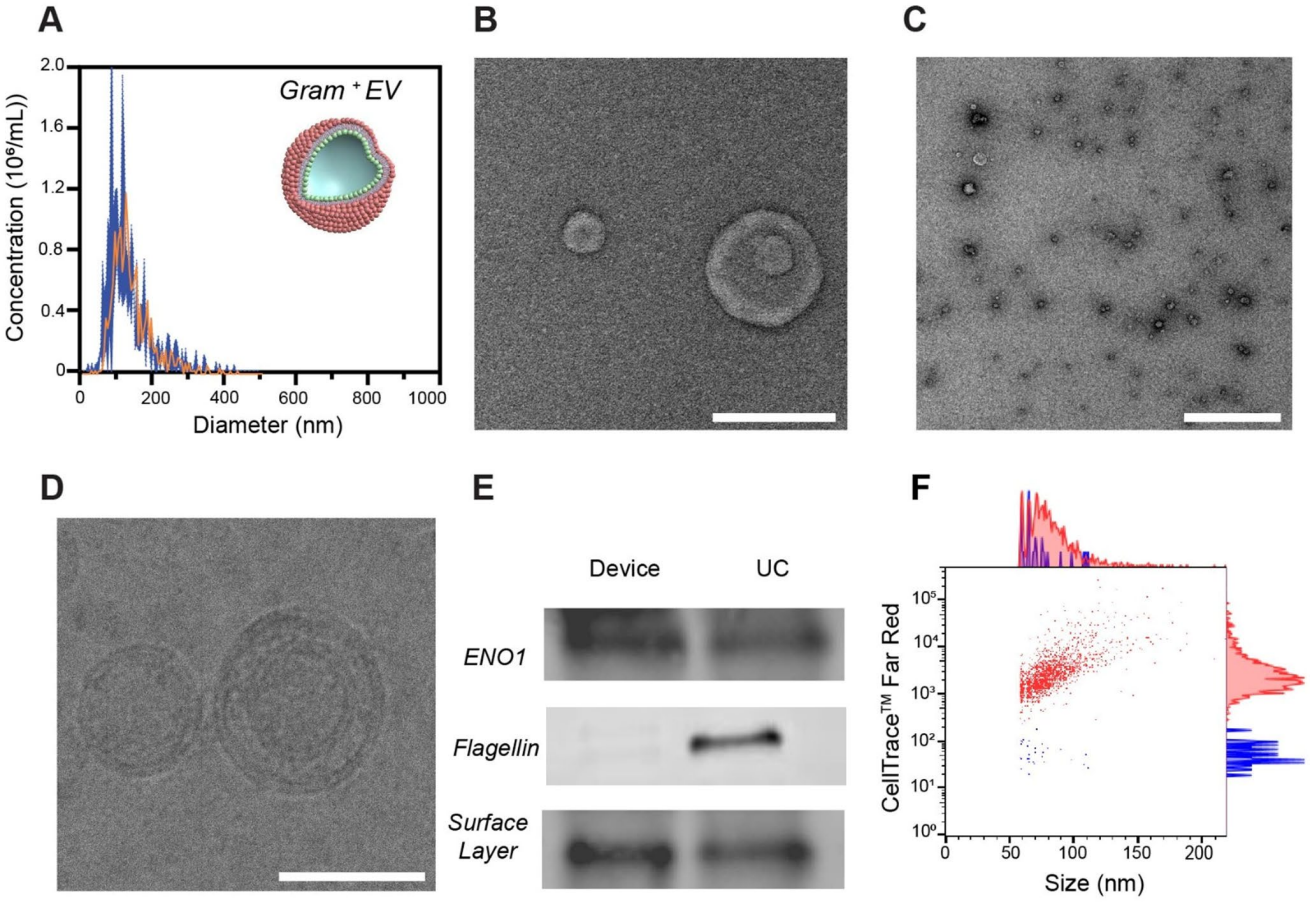
Characterization of bacterial EVs from Gram-positive *Lb. fermentum* supernatant. (**A**) NTA plot of EVs isolated using the device with an anti-S-layer antibody coating. Experimental data are expressed as mean (orange) ± SD (dark blue) (n = 3). Schematic representation of a Gram-positive EV with the cytoplasmic membrane colored in rose pink. (**B**) Representative high magnification TEM image of isolated EVs in PBS. Scale bar: 100 nm. (**C**) Representative low magnification TEM image of isolated EVs in PBS. Scale bar: 1000 nm. (**D**) Representative high magnification cryo-TEM image of two isolated EVs in PBS. Scale bar: 100 nm. (**E**) Western blot analysis of bacterial EV protein markers (ENO1, flagellin and S-layer), showing the EV proteins isolated with the device in the left lane, and those isolated by UC in the right lane. (**F**) NanoFCM analysis of CTFR stained *Lb. fermentum*-derived EVs. A representative bivariate dot plot of size (nm, x axis) vs CTFR (y axis) is shown, with corresponding histograms displayed alongside. CTFR-positive events are shown in red, while CTFR-negative events are shown in blue. A representative plot is reported, with the mean values of 84.8 ± 9.5% for CTFR-positive events and 15.2 ± 9.5% for CTFR-negative events. Original uncropped Western blots are provided in the supporting information (Fig. S6)

For both types of experiments, EVs were isolated in triplicates using either the device or UC, collected in PBS and subsequently characterized (Figs. 2 and 3, Fig. S2, S3, S5 and Table S3). Following isolation with the device, NTA revealed mean particle diameters of 107.3 ± 2.8 nm for *E. coli*-derived EVs, and 140.7 ± 10.6 nm for *Lb. fermentum*-derived EVs, with concentrations of 1.6 × 10¹¹ and 1.9 × 10⁸ particles/mL, respectively (Fig. 2A, and 3A). With UC, the mean diameters were 104.2 ± 14.7 nm for *E. coli*-derived EVs and 138.8 ± 9.4 nm for *Lb. fermentum*-derived EVs, with concentrations of 1.2 × 10¹¹ and 1.6 × 10⁹ particles/mL, respectively (Fig. S2A, S3A). Overall, the mean particle diameters were comparable between the two isolation methods, whereas the particle size distribution was notably broader in the UC-isolated samples vs. the device. This discrepancy may be due to a higher presence of contaminants, such as protein aggregates and cell debris, retained during the UC process. DLS analysis revealed comparable size values (~ 120–130 nm) for EVs isolated using the device and UC (Fig. S5A, Table S3). In contrast, the zeta potential values differed between the two methods, possibly reflecting the presence of additional impurities (Fig. S5B, Table S3). The less negative zeta potential observed in EVs isolated from *Lb. fermentum* compared to those from *E. coli* could be attributed to differences in cell wall structure and composition. EVs derived from Gram-negative bacteria, such as *E. coli*, are characterized by the presence of abundant LPS on their membranes [1, 2, 11]. LPS carry a high negative charge due to phosphate groups and other negatively charged moieties that contribute to the more negative zeta potential of *E. coli*-derived EVs. EVs from Gram-positive bacteria, such as *Lb. fermentum*, lack LPS but contain lipoteichoic acid [30]. While LTA contributes to the negative charge of *Lb. fermentum*-derived EVs, its effect is less pronounced than LPS [31]. TEM micrographs of the device- and UC-isolated EVs revealed a field of spherical vesicles (Figs. 2B and C and 3B and C, Fig. S2B, C and Fig. S3B, C), with the distinctive cup-shaped morphology observable at low magnification. The cup-shaped morphology was less pronounced in Gram-positive EVs, possibly due to their thicker outer layer or the reduced staining efficiency, which may lead to poor dehydration of the vesicles [32]. The phospholipid bilayer membrane structure was clearly visualized in cryo-TEM images (Figs. 2D and 3D, Fig. S2D). The cryo-TEM image of the *Lb. fermentum* sample collected post UC was heavily contaminated with proteins and/or cell debris, obscuring the grid and preventing clear visualization. Next, protein profiling was performed by Western blot analysis (Figs. 2E and 3E). For *E. coli*-derived EVs, the surface porins OmpA and OmpC, as well as the cytosolic protein elongation factor TU (EF-TU), were detected (Fig. 2E). The latter has the function to shuttle aminoacylated tRNAs to the ribosome during protein translation [18, 33]. Although initially predicted to be predominantly expressed in the bacterial cytoplasm, EF-TU has also been detected in OMVs from various *E. coli* strains [33, 34]. In contrast to UC, the bacterial EV sample isolated with the device barely showed any flagellin, a contaminating protein marker, suggesting higher purity of the isolated sample [18]. During bacterial culture or sample processing, small amounts of OmpA proteins may be released from cell debris or disrupted vesicles. Therefore, we performed Western blot analysis on the collected supernatant, as a control experiment. However, no detectable bands were observed for these proteins in the supernatant samples, suggesting that their concentrations were below the detection limit of Western blotting (Fig. S7).

For *Lb. fermentum*-derived EVs, Western blot analysis revealed the presence of S-layer and enolase-1 (ENO1) (Fig. 3E). ENO1 is a multifunctional protein that primarily serves as a glycolytic enzyme in the cytosol, catalyzing the conversion of 2-phospho-D-glycerate to phosphoenolpyruvate during aerobic glycolysis [35]. It is found both as a cytoplasmic and a surface-associated protein in many Gram-positive bacteria and their derived EVs [36, 37]. Unlike UC, a faint band corresponding to the contaminant flagellin was observed in the bacterial EV sample isolated using the device method. This contaminant might eventually be removed by optimizing the washing step [20]. NanoFCM was used to further validate the presence of bacterial EVs in all collected samples (Figs. 2F and 3F, Fig. S2E, F, S3D, E). To this purpose, bacterial EVs were labeled with CellTrace™ Far Red (CTFR) dye. After staining the samples isolated with the electrochemical device, 91.9 ± 2.8% of events in *E. coli*-derived EVs and 84.8 ± 9.5% of events in *Lb. fermentum*-derived EVs were positive for CTFR staining, suggesting that the majority of detected particles were EVs. Finally, a pilot classical bottom-up proteomics analysis coupled to label-free quantification of fragment spectra based on Data-Independent-Analysis (DIA) was performed to characterize the proteomes of isolated vesicles. Data were analyzed against a *Limosilactobacillus fermentum* and rabbit database, confirming no IgG was identified in the samples.

Taken together, these results confirm that EVs were isolated from the medium of *E. coli* and *Lb. fermentum* bacterial cells using our electrochemical device.

### Isolation of bacterial EVs from Gram-negative *E. coli* contaminated urine sample

**Fig. 4:**
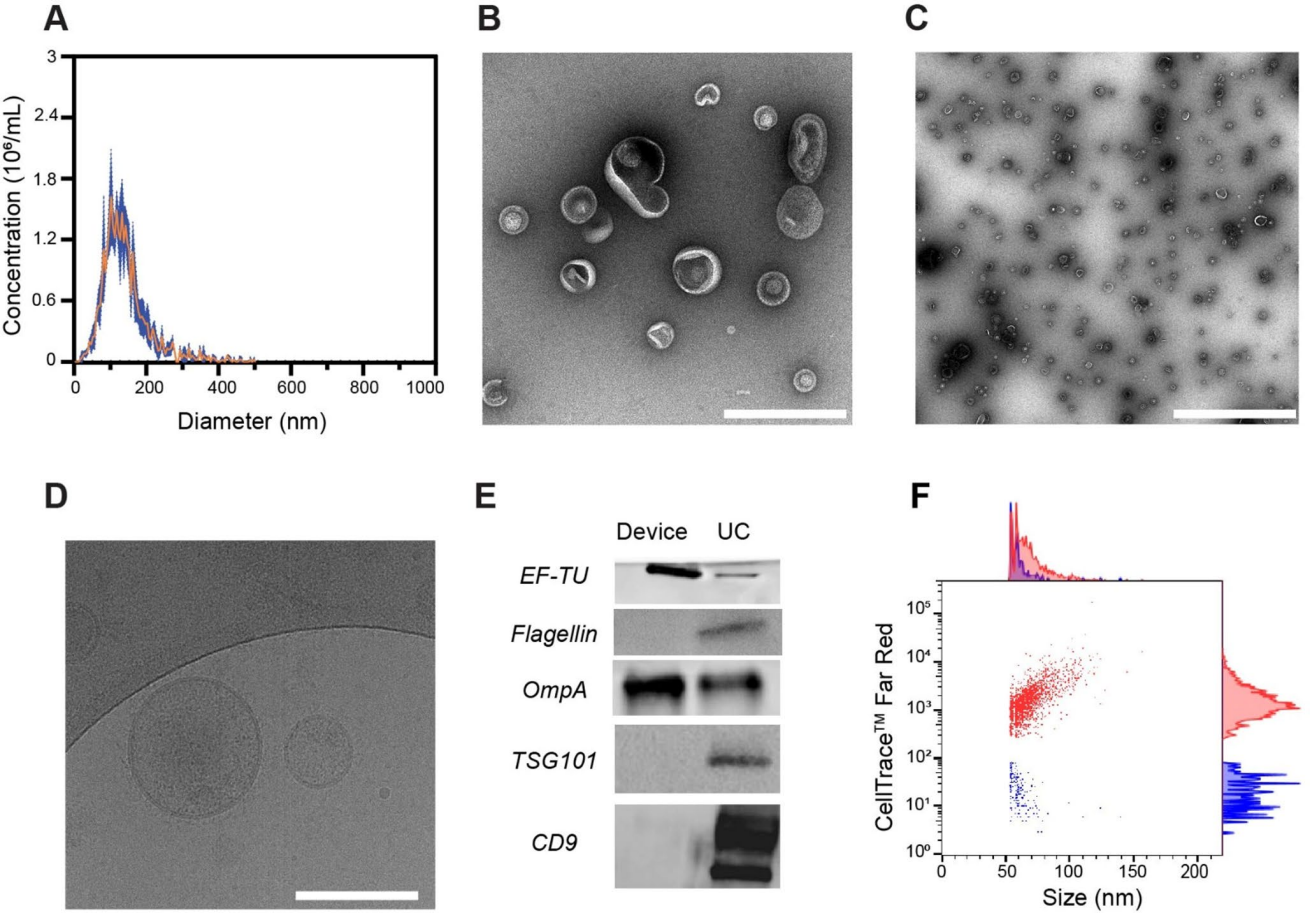
Characterization of bacterial EVs from Gram-negative *E. coli* contaminated urine sample. (**A**) NTA plot of EVs isolated from patient-derived urine samples, using the device with an anti-OmpA antibody coating. Experimental data are expressed as mean (orange) ± SD (dark blue) (n = 3). (**B**) Representative high magnification TEM image of a field of isolated EVs in PBS. Scale bar: 200 nm. (**C**) Representative low magnification TEM image of a field of isolated EVs in PBS. Scale bar: 1000 nm. (**D**) Representative high magnification cryo-TEM image of two isolated EVs in PBS. Scale bar: 100 nm. (**E**) Western blot analysis of bacterial EV protein markers (EF-TU, flagellin, OmpA), and of human EV protein markers (TSG101, CD9), showing the EV proteins isolated with the device in the left lane, and those isolated with UC in the right lane. (**F**) NanoFCM analysis of CTFR stained EVs from *E. coli* contaminated urine sample. A representative bivariate dot plot of size (nm, x axis) vs CTFR (y axis) is shown, with corresponding histograms displayed alongside. CTFR-positive events are shown in red, while CTFR-negative events are shown in blue. A representative plot is reported, with the mean values of 89.2 ± 2.8% for CTFR-positive events and 10.8 ± 2.8% for CTFR-negative events. Original uncropped Western blots are provided in the supporting information (Fig. S6)

Urinary EVs provide valuable insights into the health of the urinary system, particularly the functionality of podocytes and renal tubular cells [38]. Urine, which can be collected easily, frequently, and in large quantities through noninvasive methods, is an ideal fluid for biomarker analysis [38]. However, its complex composition—including proteins, salts, urea, and various metabolites—can dilute EVs and obscure key biomarkers. *E. coli*, a bacterium normally residing in the gastrointestinal tract, can migrate into the urinary system and cause UTIs [24]. Although there exist established diagnostic methods for UTIs, isolating bacterial EVs could provide complementary information, offering deeper insights into infection mechanisms and disease progression. As a proof of concept, we evaluated the device for its ability to isolate *E. coli-*derived EVs from urine (Fig. 4). *E. coli* was spiked into the urine sample to achieve a concentration of 10 CFU/mL, which falls within the typical bacterial load associated with UTIs [24]. The samples were processed with the device or UC and analyzed as described above (Fig. 4, S4, S5, Table S3). As for the isolation of vesicles from the culture medium, EVs collected with the device from urine had an average diameter of 126.2 ± 1.8 nm and 124.6 ± 4.5 nm, as shown by NTA and DLS, respectively (Fig. 4, Fig. S5 and Table S3). Instead, EVs isolated with UC from urine showed an average diameter of 145.2 ± 6.0 nm and 175 ± 30.2 nm, as reported by NTA and DLS, respectively, (Fig. S4, Fig. S5 and Table S3). The particle concentration was found to be 2.8 × 10^10^ particles/mL, similar to the concentration value of 3.3 × 10^10^ particles/mL after isolation with UC. NanoFCM also revealed a mean value of 89.2 ± 2.8% of the events to be positive for CTFR, while a mean value of 10.8 ± 2.8% to be negative for CTFR (Fig. 4).

Similar to the results from the cell culture media, the device outperformed UC in efficiently isolating EVs. Western blot analysis of the samples (Fig. 4E) indeed revealed that, in contrast to UC, which co-isolated both human and bacterial EVs from contaminated urine samples (detecting human EV markers such as TSG101 and CD9 alongside bacterial markers like EF-TU and OmpA), the electrochemical device was more selective, with reduced contamination from human EVs and the flagellin marker. This feature is essential for clinical diagnostics, as the ability to distinguish between bacterial and host EVs can provide valuable insights into the diagnosis and progression of infections [39].

### Isolated *Lactobacillus fermentum*-derived EVs activate TLR4 pathway

**Fig. 5:**
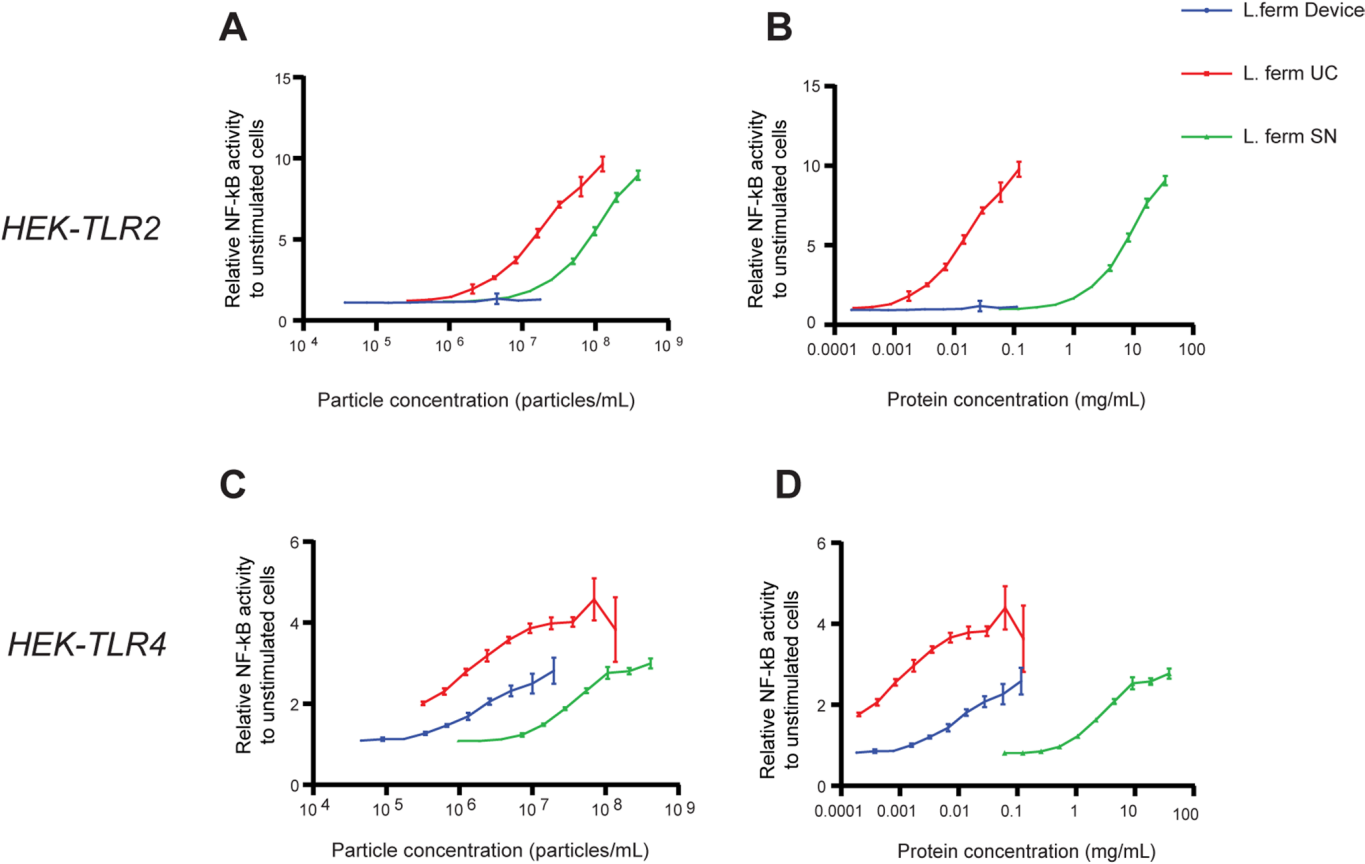
Assessment of NF-κB activity in HEK-TLR2 and HEK-TLR4 reporter cells in response to *Lb. fermentum* I3929 bacterial EVs, as a function of: (**A**) EV particle concentration (particles/mL) and (**B**) EV protein concentration (mg/mL) in HEK-TLR2 cells; (**C**) EV particle concentration (particles/mL) and (**D**) EV protein concentration (mg/mL) in HEK-TLR4 cells. Reporter cells were stimulated for 24 h at 37 °C. Mean ± SD (*n* = 4, technical replicates), SN = *Lb. fermentum* supernatant

Lactic acid-producing bacteria are widely recognized for their probiotic benefits, including bolstering the host’s immune system, enhancing feed digestibility, and mitigating metabolic disorders [22, 23]. *Lb. fermentum* is a Gram-positive bacterium within the *Lactobacillus* genus, known for its ability to boost immune responses and help prevent gastrointestinal and upper respiratory infections [22, 23]. In a pilot experiment, we evaluated whether the bacterial EVs isolated from *Lb. fermentum* supernatant, using the PMED and UC, were able to selectively modify the expression of negative regulators of Toll-like receptor 4 or 2 (TLR4 or TLR2) signaling in HEK-TLR4 and HEK-TLR2 reporter cell lines, respectively. To this end, the cells were incubated with UC- or device-isolated EVs, or native fermentation supernatant for 24 h. NF-kB activation was quantified by measurement of the secreted embryonic alkaline phosphatase (SEAP) activity (Fig. 5). *Lb. fermentum* fermentation supernatant was shown to induce TLR4 and TLR2 signaling in HEK-TLR4 and HEK-TLR2 reporter cell lines, respectively, indicating the presence of TLR2 and TLR4 agonists secreted by *Lb. fermentum* during fermentation (Fig. 5).

The bacterial EVs collected with the device did not induce activation of TLR2 (Fig. 5A-B), yet they were capable of stimulating TLR4 in this HEK reporter system (Fig. 5C-D). In contrast, the UC-isolated EVs indiscriminately activated both TLR4 and TLR2, more potently than the native *Lb. fermentum* supernatant. This differential activation may stem from the absence of TLR2-activating components in device-isolated EVs, whereas UC isolation likely retains additional bacterial fragments that also engage TLR2. In this respect, in Gram-positive bacteria, TLR2-mediated recognition of PAMPs, such as LTA and PGN, triggers downstream signaling through MyD88, ultimately leading to NF-κB activation. The absence of TLR2 activation in device-isolated EVs suggests that the composition of the EV subset preferentially supports TLR4 engagement and/or LTA and PGN are lower in content compared to UC [40]. Proteomics and lipidomics analyses would, in the future, be essential to uncover the compositional differences between bacterial EV samples isolated using the device vs. UC.

Overall, our results highlight the ability of the PMED in isolating functionally distinct bacterial EV populations, providing a more targeted approach to studying immune responses.

## Conclusion

In this study, EVs from Gram-negative (*E. coli*) and Gram-positive (*Lb. fermentum*) bacterial supernatants were isolated with an electrochemically controlled device. This device addresses key limitations of traditional methods like UC, such as contamination and limited selectivity. By combining immunoaffinity-based EV capture with voltage-driven release, the device achieves enhanced selectivity, and reduced contaminats compared to conventional techniques. This isolation method creates avenues for biomarker discovery in liquid diagnostics and the targeted modulation of immune responses. Furthermore, the versatile antibody-conjugation strategy broadens the device’s applicability to a wide range of bacterial and non-bacterial sources. For instance, beyond the previously reported isolation of mammalian EVs [20], future studies could explore its use in isolating EVs from plant-derived samples. Such applications may provide insights into plant-microbe interactions, crop improvement strategies, or the role of plant EVs in stress responses.

## Supplementary Information

Below is the link to the electronic supplementary material.

Supplementary Material 1

## Data Availability

The data supporting the findings of this study will be available in the ETH Research Collection at https://www.research-collection.ethz.ch.

## References

[1] Schwechheimer C, Kuehn MJ. Outer-membrane vesicles from Gram-negative bacteria: biogenesis and functions. Nat Rev Microbiol. 2015;13:605–19. 10.1038/nrmicro3525. 26373371 10.1038/nrmicro3525 PMC5308417

[2] Hosseini-Giv N, Basas A, Hicks C, El-Omar E, El-Assaad F, Beheshti EH. Bacterial extracellular vesicles and their novel therapeutic applications in health and cancer. Front Cell Infect Microbiol. 2022;12:962216. 10.3389/fcimb.2022.962216. 36439225 10.3389/fcimb.2022.962216 PMC9691856

[3] Mandal PK, Ballerin G, Nolan LM, Petty NK, Whitchurch CB. Bacteriophage infection of Escherichia coli leads to the formation of membrane vesicles via both explosive cell Lysis and membrane blebbing. Microbiol. 2021;167:001021. 10.1099/mic.0.001021. 10.1099/mic.0.001021 PMC8289217 33871329

[4] Junhua X, Qiqiong L, Nie S. Bacterial extracellular vesicles: an emerging postbiotic. Trends Food Sci Technol. 2024;143:104275. 10.1016/j.tifs.2023.104275.

[5] Bose S, Aggarwal S, Singh DV, Acharya N. Extracellular vesicles: an emerging platform in gram-positive bacteria. Microb Cell. 2020;7:312. 10.15698/mic2020.12.737. 33335921 10.15698/mic2020.12.737 PMC7713254

[6] Bitto NJ, Chapman R, Pidot S, Costin A, Lo C, Choi J, D’Cruze T, Reynolds EC, Dashper SG, Turnbull L, Whitchurch CB, Stinear TP, Stacey KJ, Ferrero RL. Bacterial membrane vesicles transport their DNA cargo into host cells. Sci Rep. 2017;7:7072. 10.1038/s41598-017-07288-4. 28765539 10.1038/s41598-017-07288-4 PMC5539193

[7] Kumari P, Wright SS, Rathinam AV. Role of extracellular vesicles in immunity and host defense. Immunol Invest. 2024;53:10–25. 38348776 10.1080/08820139.2024.2312896 PMC11111308

[8] Wu Q, Li Z, Kan J, Liu Q, Fu C, Zhang Y, Liu X, Du J. Insights into the unique roles of extracellular vesicles for gut health modulation: mechanisms, challenges, and perspectives. Curr Res Microb Sci. 2024;7:100301. 10.1016/j.crmicr.2024.100301. 39525958 10.1016/j.crmicr.2024.100301 PMC11550031

[9] Molina-Tijeras AJ, Gálvez J, Rodríguez-Cabezas ME. The Immunomodulatory properties of extracellular vesicles derived from probiotics: A novel approach for the management of Gastrointestinal diseases. Nutr. 2019;11:1038. 10.3390/nu11051038. 10.3390/nu11051038 PMC6567093 31075872

[10] Asong J, Wolfert MA, Maiti KK, Miller D, Boons GJ. Binding and cellular activation studies reveal that Toll-like receptor 2 can differentially recognize peptidoglycan from Gram-positive and Gram-negative bacteria. JBC. 2009;284:8643–53. 10.1074/jbc.M806633200. 10.1074/jbc.M806633200 PMC2659223 19164296

[11] Peregrino ES, Castañeda-Casimiro J, Vázquez-Flores L, Estrada-Parra S, Wong-Baeza C, López JS, Wong-Baeza I. The role of bacterial extracellular vesicles in the immune response to pathogens, and therapeutic opportunities. Int J Mol Sci. 2024;25:6210. 10.3390/ijms25116210. 38892397 10.3390/ijms25116210 PMC11172497

[12] Li M, Lee K, Hsu M, Nau G, Mylonakis E, Ramratnam B. Lactobacillus-derived extracellular vesicles enhance host immune responses against vancomycin-resistant enterococci. BMC Microbiol. 2017;17:1–8. 10.1186/s12866-017-0977-7. 28288575 10.1186/s12866-017-0977-7 PMC5348868

[13] Park JY, Kang CS, Seo HC, Shin JC, Kym SM, Park YS, Shin TS, Kim JG, Kim YK. Bacteria-Derived extracellular vesicles in urine as a novel biomarker for gastric cancer: integration of liquid biopsy and metagenome analysis. Cancers. 2021;13:4687. 10.3390/cancers13184687. 34572913 10.3390/cancers13184687 PMC8468964

[14] Moore KA, Petersen AP, Zierden HC. Microorganism-derived extracellular vesicles: emerging contributors to female reproductive health. Nanoscale. 2024;16:8216. 10.1039/d3nr05524h. 38572613 10.1039/d3nr05524h

[15] Luo ZW, Xia K, Liu YW, Liu JH, Rao SS, Hu XK, Chen CY, Xu R, Wang ZX, Xie H. Extracellular vesicles from Akkermansia muciniphila elicit antitumor immunity against prostate cancer via modulation of CD8 + T cells and macrophages. Int J Nanomed. 2021;2949–63. 10.2147/IJN.S304515. 10.2147/IJN.S304515 PMC8068512 33907401

[16] Grange C, Dalmasso A, Cortez JJ, Spokeviciute B, Bussolati B. Exploring the role of urinary extracellular vesicles in kidney physiology, aging, and disease progression. Am J Physiol Cell Physiol. 2023;325:C1439–50. 10.1152/ajpcell.00349.2023. 37842748 10.1152/ajpcell.00349.2023 PMC10861146

[17] Mizutani K, Kawakami K, Horie K, Fujita Y, Kameyama K, Kato T, Nakane K, Tsuchiya T, Yasuda M, Masunaga K, Kasuya Y, Masuda Y, Deguchi T, Koie T, Ito M. Urinary exosome as a potential biomarker for urinary tract infection. Cell Microbiol. 2019: 21; e13020. 10.1111/cmi.13020 30817089

[18] Tulkens J, De Wever O, Hendrix A. Analyzing bacterial extracellular vesicles in human body fluids by orthogonal biophysical separation and biochemical characterization. Nat Protoc. 2020;15:40–67. 10.1038/s41596-019-0236-5. 31776460 10.1038/s41596-019-0236-5

[19] Simonsen JB. What are we looking at?? Extracellular vesicles, lipoproteins, or both?? Circ Res. 2017;121:920–2. 10.1161/CIRCRESAHA.117.311767. 28963190 10.1161/CIRCRESAHA.117.311767

[20] Krivitsky V, Krivitsky A, Mantella V, Greenwald MBY, Sankar DS, Betschmann J, Bader J, Zoratto N, Schreier K, Feiss S, Walker D, Dengjel J, Werner S, Leroux JC. Ultrafast and controlled capturing, loading, and release of extracellular vesicles by a portable microstructured electrochemical fluidic device. Adv Mat. 2023;35:2212000. 10.1002/adma.202212000. 10.1002/adma.202212000 37452635

[21] Tian C, Wang K, Zhao M, Cong S, Di X, Li R. Extracellular vesicles participate in the pathogenesis of sepsis. Front Cell Infect Microbiol. 2022;12:1018692. 10.3389/fcimb.2022.1018692. 36579343 10.3389/fcimb.2022.1018692 PMC9791067

[22] Rodrıguez-Nogales A, Algieri F, Garrido-Mesa J, Vezza T, Pilar Utrilla M, Chueca N, Garcia F, Olivares M, Rodrıguez-Cabezas ME, Galvez J. Differential intestinal anti-inflammatory effects of Lactobacillus fermentum and Lactobacillus salivarius in DSS mouse colitis: impact on MicroRNAs expression and microbiota composition. Mol Nutr Food Res. 2017;61:1700144. 10.1002/mnfr.201700144. 10.1002/mnfr.201700144 28752563

[23] Naghmouchia K, Belguesmia Y, Bendali F, Spano G, Seal BS, Drider D. Lactobacillus fermentum: a bacterial species with potential for food preservation and biomedical applications. Crit Rev Food Sci Nutr. 2020;20:3387–99. 10.1080/10408398.2019.1688250. 10.1080/10408398.2019.1688250 31729242

[24] Drage LKL, Robson W, Mowbray C, Ali A, Perry JD, Walton KE, Harding C, Pickard R, Hall J, Aldridge PD. Elevated urine IL-10 concentrations associate with Escherichia coli persistence in older patients susceptible to recurrent urinary tract infections. Immun Ageing. 2019;16:1–11. 10.1186/s12979-019-0156-9. 31338112 10.1186/s12979-019-0156-9 PMC6625057

[25] Nečas D, Klapetek P. Gwyddion: an open-source software for SPM data analysis. Open Phys. 2012;10:181–8. 10.2478/s11534-011-0096-2.

[26] Bader J, Rüedi P, Mantella V, Geisshüsler S, Brigger F, Qureshi BM, Ortega Arroyo J, Montanari E, Leroux JC. Loading of extracellular vesicles with nucleic acids via hybridization with Non-Lamellar liquid crystalline lipid nanoparticles. Adv Sci. 2025;12:2404860. 10.1002/advs.202404860. 10.1002/advs.202404860 PMC11848734 39741121

[27] Zhou G, Wang Q, Wang Y, Wen X, Peng H, Peng R, Shi Q, Xie X, Li L. Outer membrane porins contribute to antimicrobial resistance in Gram-Negative bacteria. Microorg. 2023;11:1690. 10.3390/microorganisms11071690. 10.3390/microorganisms11071690 PMC10385648 37512863

[28] Nie D, Hu Y, Chen Z, Li M, Hou Z, Luo X, Mao X, Xue X. Outer membrane protein A (OmpA) as a potential therapeutic target for acinetobacter baumannii infection. J Biomed Sci. 2020;27:1–8. 10.1186/s12929-020-0617-7. 31954394 10.1186/s12929-020-0617-7 PMC6969976

[29] Palomino MM, Allievi MC, Gordillo TB, Bockor SS, Martin JF, Ruzal SM. Surface layer proteins in species of the family lactobacillaceae. Microb Biotechnol. 2023;16:1232–49. 10.1111/1751-7915.14230. 36752119 10.1111/1751-7915.14230 PMC10221546

[30] Jacobson KH, Gunsolos IL, Kuech TR, Troiano JM, Melby ES, Lohse SE, Hu D, Chrisler WB, Murphy CJ, Orr G, Geiger FM, Haynes CL, Pedersen JA. Lipopolysaccharide density and structure govern the extent and distance of nanoparticle interaction with actual and model bacterial outer membranes. Environ Sci Technol. 2015;49:10642–50. 10.1021/acs.est.5b01841. 26207769 10.1021/acs.est.5b01841 PMC4643684

[31] Pradhan D, Gulati G, Avadhani R, Rashmi HM, Soumya K, Kumari A, Gupta A, Dwivedi D, Kaushik JK, Grover S. Postbiotic Lipoteichoic acid of probiotic Lactobacillus origin ameliorates inflammation in HT-29 cells and colitis mice. Int J Biol Macromol. 2023;236:123962. 10.1016/j.ijbiomac.2023.123962. 36907160 10.1016/j.ijbiomac.2023.123962

[32] Chuo STY, Chien JCY, Lai CPK. Imaging extracellular vesicles current and emerging methods. J Biomed Sci. 2018;25:1–10. 10.1186/s12929-018-0494-5. 30580764 10.1186/s12929-018-0494-5 PMC6304785

[33] Harvey KL, Jarocki VM, Charles IG, Djordjevic SP. The diverse functional roles of elongation factor Tu (EF-Tu) in microbial pathogenesis. Front Microbiol. 2019;10:2351. 10.3389/fmicb.2019.02351. 31708880 10.3389/fmicb.2019.02351 PMC6822514

[34] Torres AN, Veloso NC, Costa P, Cádiz L, Del Canto F, Venegas SA, Nitsche ML, Coloma-Rivero RF, Montero DA, Vida RM. Deciphering additional roles for the EF-Tu, l-Asparaginase II and ompt proteins of Shiga Toxin-Producing Escherichia coli. Microorganisms. 2020;8:1184. 10.3390/microorganisms8081184. 32759661 10.3390/microorganisms8081184 PMC7464798

[35] Chung IC, Huang WC, Huang YTS, Chen ML, Tsai AW, Wu PY, Yuan TT. Unrevealed roles of extracellular enolase–1 (ENO1) in promoting Glycolysis and pro–cancer activities in multiple myeloma via hypoxia–inducible factor 1α. Oncol Rep. 2023;50:205. 10.3892/or.2023.8642. 37800625 10.3892/or.2023.8642 PMC10568254

[36] Krzyzek P, Marinacci B, Vitale I, Grande R. Extracellular vesicles of probiotics: shedding light on the biological activity and future applications. Pharmaceutics.2023;15: 522. 10.3390/pharmaceutics15020522 10.3390/pharmaceutics15020522 PMC9967243 36839844

[37] Antikainen J, Kuparinen V, Lähteenmäki K, Korhonen TK. Enolases from Gram-positive bacterial pathogens and commensal lactobacilli share functional similarity in virulence-associated traits. FEMS Immunol Med Microbiol. 2007;51:526–34. 10.1111/j.1574-695X.2007.00330.x. 17892475 10.1111/j.1574-695X.2007.00330.x

[38] Gámez-Valero A, Lozano-Ramos SI, Bancu I, Lauzurica-Valdemoros R, Borràs FE. Urinary extracellular vesicles as source of biomarkers in kidney diseases. Front Immunol. 2015;6:6. 10.3389/fimmu.2015.00006. 25688242 10.3389/fimmu.2015.00006 PMC4311634

[39] Ware JP, Shea DK, Nicholas SL, Stimson EA, Riesterer JL, Ibsen SD. Recovery and analysis of bacterial membrane vesicle nanoparticles from human plasma using dielectrophoresis. Biosensors. 2024;14:456. 10.3390/bios14100456. 39451671 10.3390/bios14100456 PMC11505931

[40] Ribeiro CMS, Hermsen T, Taverne-Thiele AJ, Savelkoul HFJ, Wiegertjes GF. Evolution of recognition of ligands from Gram-Positive bacteria: similarities and differences in the TLR2-Mediated response between mammalian vertebrates and teleost fish. J Immunol. 2010;184:2355–68. 10.4049/jimmunol.0900990. 20118281 10.4049/jimmunol.0900990

