# Supplementary material for: Isolation of bacterial extracellular vesicles from raw samples using a portable microstructured electrochemical device
Source: Drug Deliv Transl Res. 2025 Aug 26;16(5):1479–94. doi: 10.1007/s13346-025-01954-1 (PMC13038669; doi:10.1007/s13346-025-01954-1)
Supplement: Supplementary file 1 — Supplementary Material 1 [file 13346_2025_1954_MOESM1_ESM.pdf]

Isolation of Bacterial Extracellular Vesicles from Raw Samples Using a Portable Microstructured Electrochemical Device

Valeria Mantella[1] · Siiri Bienz[2] · Finn Brigger[1] · Edouard Baulier[3] · Marie Ramus[3] · Nicole Zoratto[1] · Steffen Honrath[1] · Kumar Naresh[2] · Sibilla Sander[4] · Jörn Dengjel[4] · Renato Zenobi[2] · Vadim Krivitsky[5*] · Jean-Christophe Leroux[1*]

[1] Laboratory of Drug Formulation and Delivery, Institute of Pharmaceutical Sciences, Department of Chemistry and Applied Biosciences, ETH Zürich, Zürich 8093, Switzerland

[2] Laboratory of Organic Chemistry, Institute of Pharmaceutical Sciences, Department of Chemistry and Applied Biosciences, ETH Zürich, Zürich 8093, Switzerland

[3] OM Pharma SA, Meyrin, Geneva 1217, Switzerland

[4] Department of Biology, University of Fribourg, Chemin du Musée 10, 1700 Fribourg, Switzerland

[5] Acytronix GmbH, Wagistrasse 18, 8952 Schlieren, Switzerland

* Corresponding authors: jleroux@ethz.ch, vadimkrivitsky@acytronix.ch

Atomic Force Microscopy (AFM) imaging of the antibody-vesicle interaction

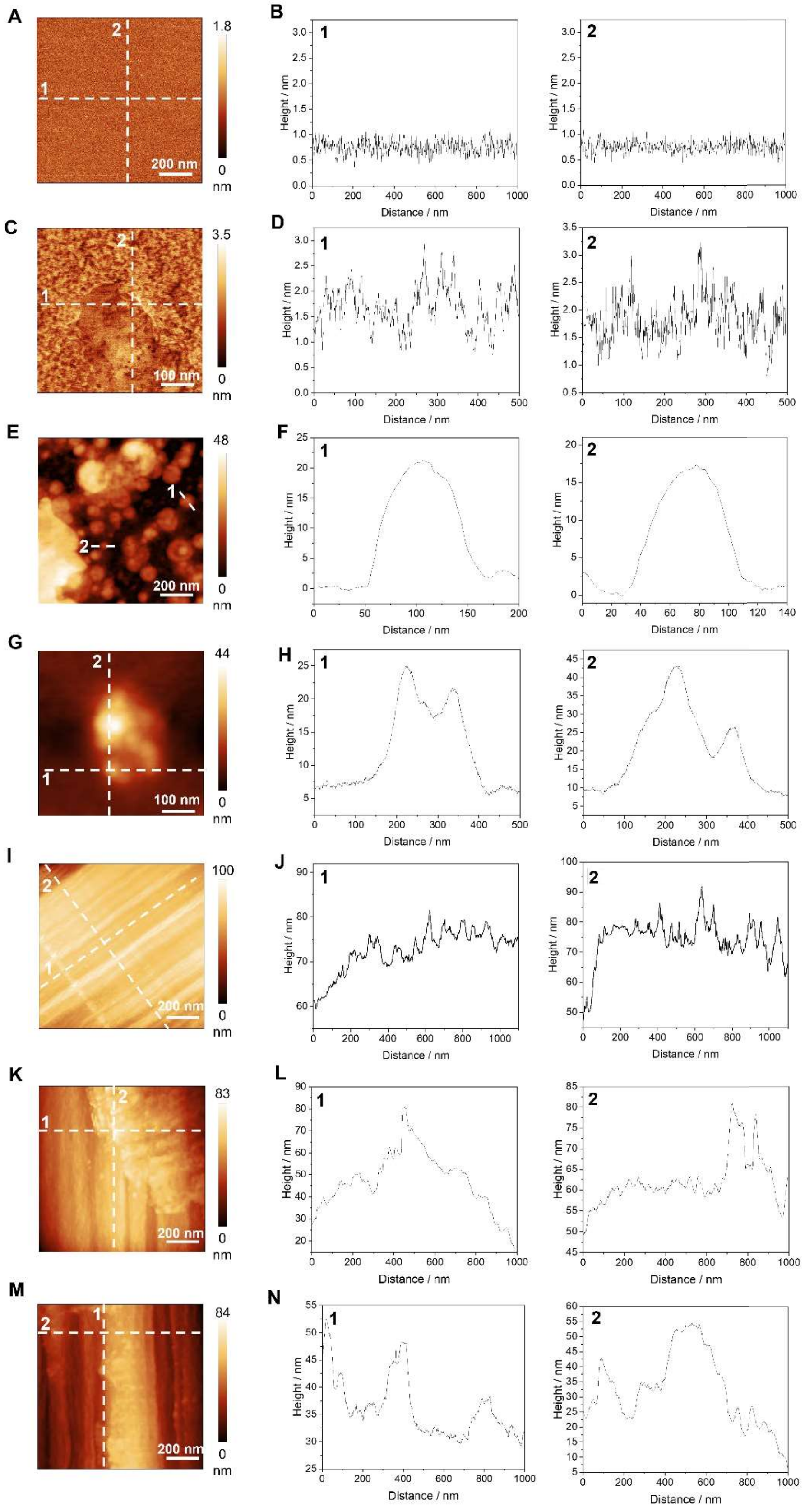
A
B
C
D
E
F
G
H
I
J
K
L
M
N
1
2
200 nm
100 nm
1.8
3.5
48
44
100
83
84
0
nm
Height / nm
Distance / nm

Fig. S1 (A) AFM topography image of a pristine mica substrate. (B) Height profiles along the lines marked in Panel A. The root mean square (RMS) roughness of the pristine mica substrate is 0.14 nm. (C) AFM topography image of the pristine antibody (anti-OmpA) sample deposited on mica substrate. (D) Height profiles along the lines marked in Panel C. The RMS roughness of the anti-OmpA is 0.40 nm. (E) AFM topography image of *E. coli*-derived EVs on mica substrate. The *E. coli*-derived EVs were separated using UC, (Section "General protocol for isolation by UC"). (F) Height profiles along the lines marked in Panel E. (G) AFM topography image of the *E. coli*-derived EV-antibody (anti-OmpA) complexes on mica substrate. (H) Height profiles along the lines marked in Panel G. (I) AFM topography image of the pristine carbon fiber surface. (J) Height profiles along the lines marked in Panel I. The RMS roughness of the pristine fiber is 8.4 nm. (K) and (M) AFM topography images of a carbon fiber incubated with EV-protein complexes, as depicted in Figure 1 in the main text. (L) and (N) Height profiles along the lines marked in Panel K and M. The RMS of the incubated carbon fibers is 14.0 nm and 12.1 nm, respectively

Fig. S1A-N present AFM topography images of the pristine mica substrate, pure antibody (anti-OmpA), isolated *E. coli*-derived EVs, the *E. coli*-derived EV-antibody (anti-OmpA) complex, pristine carbon fiber, and two additional images of a carbon fiber incubated with the EV-protein complex, along with their height profiles. Typically, the height of isolated EVs ranged from 10 – 30 nm (Fig. S1E), which is consistent with previous reports [1,2]. The 0.26 nm difference between the RMS roughness of the pristine (Fig. S1A) and protein-covered (Fig. S1C) mica substrates indicates that the protein molecules are significantly smaller than the EVs. Consequently, the height of the EV-protein complexes (Fig. S1G) was similar to the pure EVs (Figure S1E). Furthermore, the topography image of the pristine carbon fiber in Fig. S1I showed absence of any globular features like the ones clearly visible in Fig. 1F and G and Fig. S1K and S1M. The RMS roughness of the incubated carbon fibers ranged from 12.1 nm to 17.7 nm, notably exceeding that of the pristine carbon fibers (8.4 nm), indicating the successful capture of EV-protein complexes by the incubated fibers.

Isolation of bacterial EVs from Gram-negative *E. coli* and Gram-positive *Lb. fermentum* and from Gram-negative *E. coli* urine- contaminated samples with UC

EVs were isolated from 25 mL of: *E. coli* supernatant, *Lb. fermentum* supernatant and *E. coli*-contaminated urine sample, following the protocol in Section "General protocol for isolation by UC". The pellets were dispersed in 1 mL of PBS and stored at 4 °C prior to characterization.

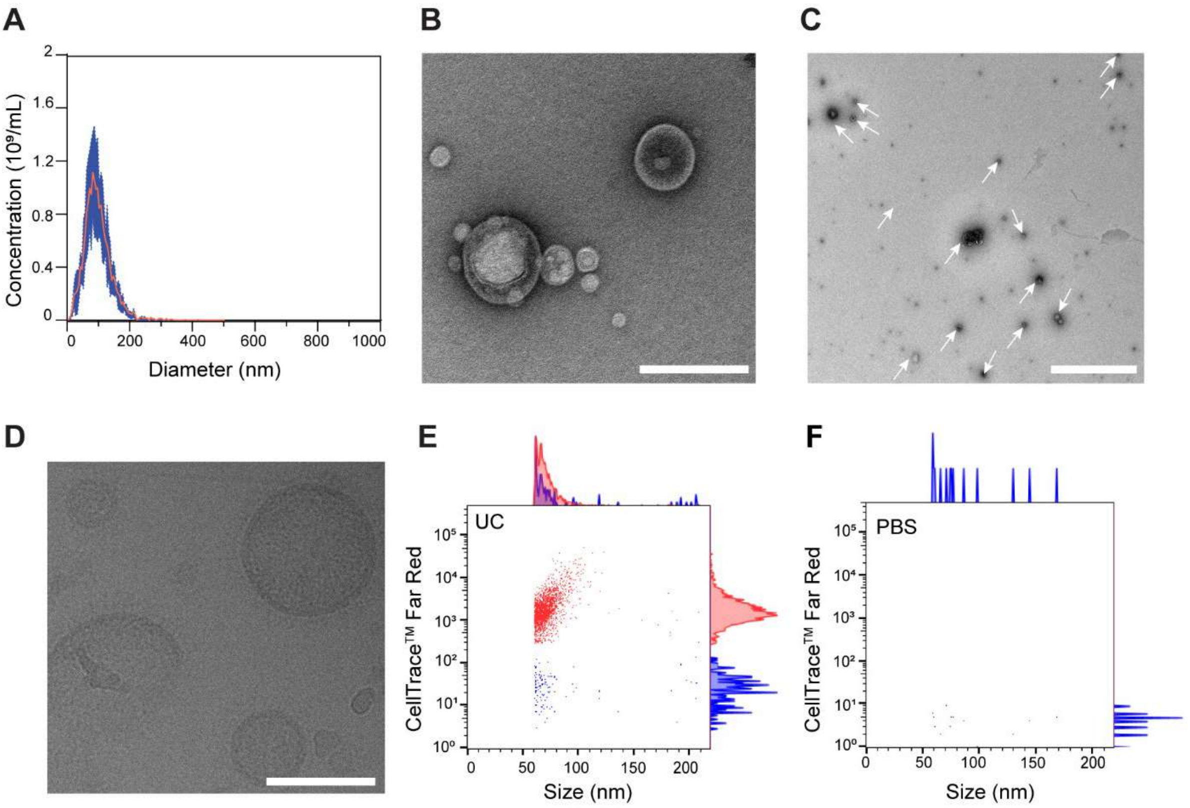

Fig. S2 Characterization of bacterial EVs isolated from Gram-negative *E. coli* supernatant, using UC. (A) NTA plot. Experimental data are expressed as mean (orange) ± SD (dark blue) (n = 3). (B) Representative low magnification TEM image of a field of isolated EVs in PBS. Scale bar: 100 nm. (C) Representative high magnification TEM image of a field of isolated EVs in PBS. Scale bar: 1000 nm. The EVs are indicated with white arrows. (D) Representative low magnification cryo-TEM image of two isolated EVs in PBS. Scale bar: 100 nm. (E) NanoFCM analysis of CellTrace™ Far Red (CTFR) stained EVs and (F) PBS control sample (buffer only). A representative bivariate dot plot of size (nm, x axis) *vs*. CTFR (y axis) is shown, with corresponding histograms displayed alongside. CTFR-positive events are shown in red, while CTFR-negative events are shown in blue. A representative plot is shown, with the mean values of size being 86.7 ± 12.0% for CTFR-positive and 13.3 ± 12.0% for CTFR-negative events. For the PBS plot, the mean values are 0% for CTFR-positive and 100% for CTFR-negative events.

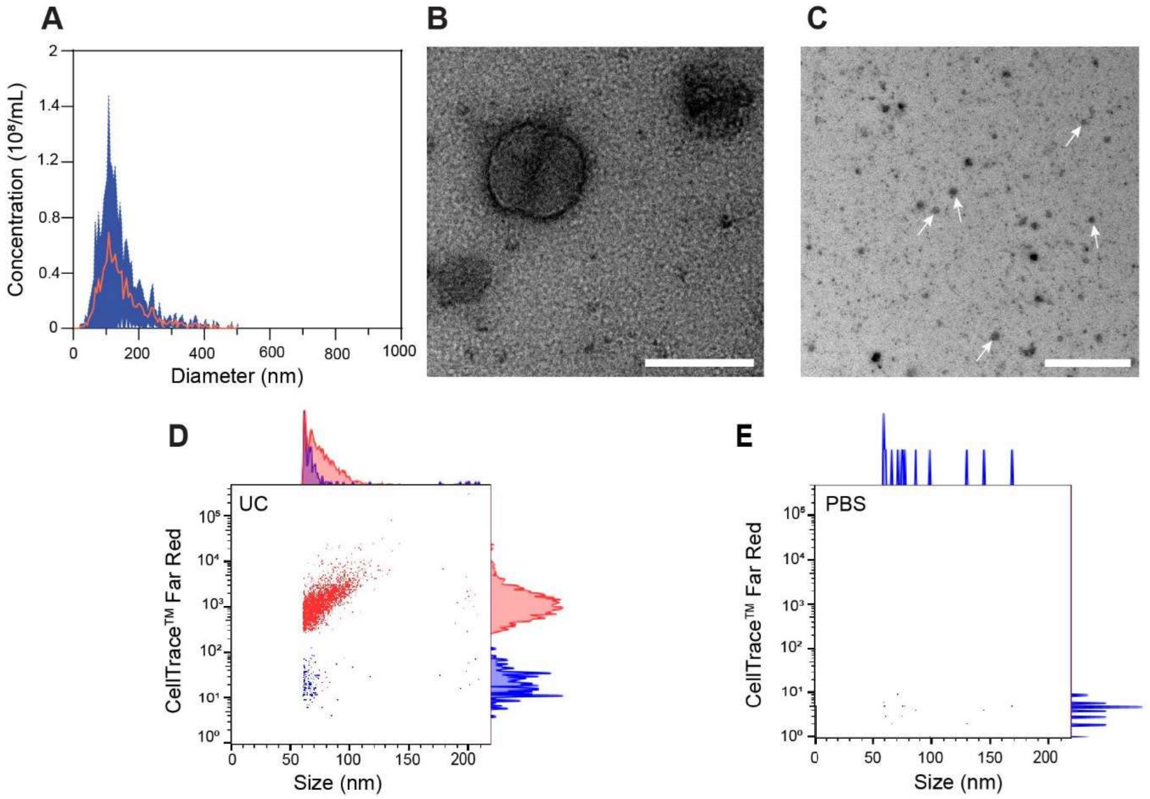

Fig. S3 Characterization of bacterial EVs isolated from *Lb. fermentum* supernatant, using UC. (A) NTA plot. Experimental data are expressed as mean (orange) ± SD (dark blue) (n = 3). (B) Representative low magnification TEM image of a field of isolated EVs in PBS. Scale bar: 200 nm. (C) Representative high magnification TEM image of a field of isolated EVs in PBS. Scale bar: 500 nm. The EVs are indicated with white arrows. (D) NanoFCM analysis of CellTrace™ Far Red (CTFR) stained EVs and (E) PBS control sample (buffer only). A representative bivariate dot plot of size (nm, x axis) *vs*. CTFR (y axis) is shown, with corresponding histograms displayed alongside. CTFR-positive events are shown in red, while CTFR-negative events are shown in blue. A representative plot is shown, with the mean values of size being 93.8 ± 0.8% for CTFR-positive and 6.2 ± 0.8% for CTFR-negative events. For the PBS plot, the mean values are 0% for CTFR-positive and 100% for CTFR-negative events.

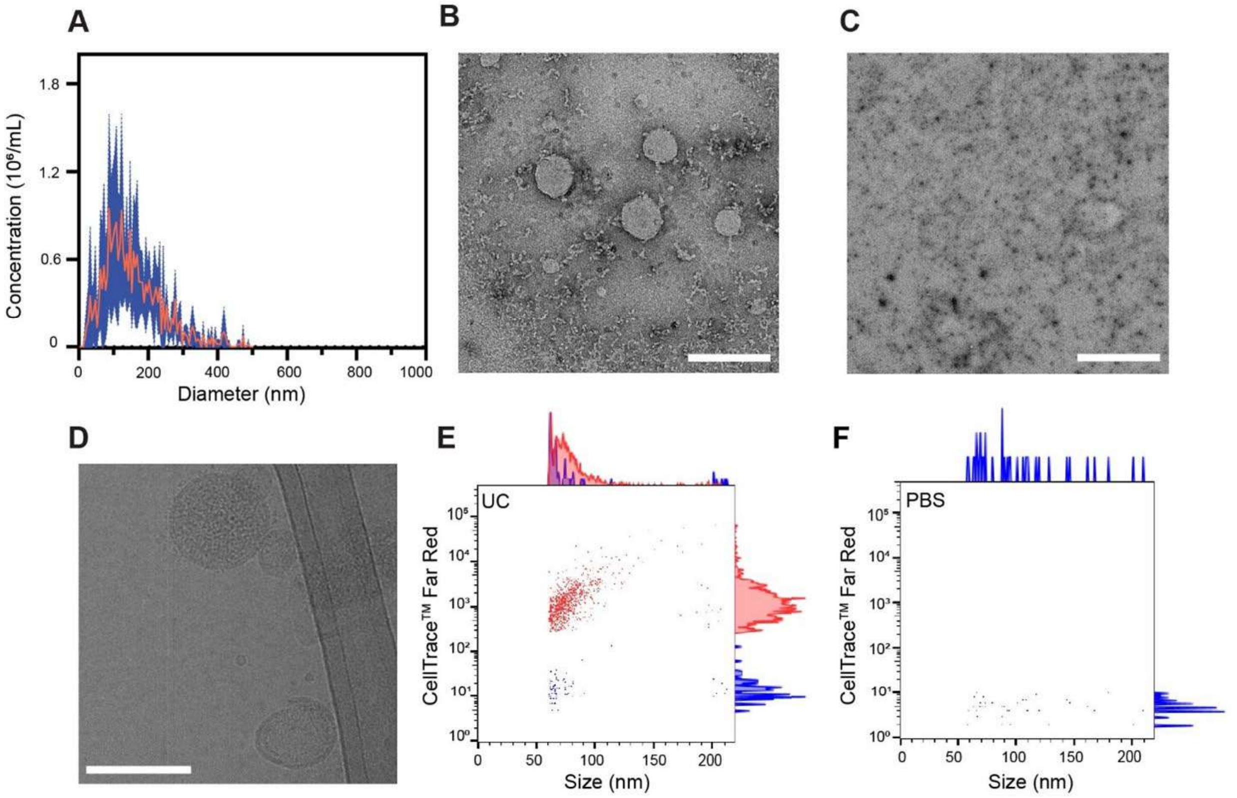

Fig. S4 Characterization of bacterial EVs isolated from Gram-negative *E. coli*-contaminated urine sample, using UC. (A) NTA plot. Experimental data are expressed as mean (orange) ± SD (dark blue) (n = 3). (B) Representative low magnification TEM image of a field of isolated EVs in PBS. Scale bar: 200 nm. (C) Representative high magnification TEM image of a field of isolated EVs in PBS. Scale bar: 1000 nm. The EVs are indicated with white arrows. (D) Representative low magnification cryo-TEM image of two isolated EVs in PBS. Scale bar: 100 nm. (E) NanoFCM analysis of CellTrace™ Far Red (CTFR) stained EVs and (E) PBS control sample (buffer only). A representative bivariate dot plot of size (nm, x axis) *vs*. CTFR (y axis) is shown, with corresponding histograms displayed alongside. CTFR-positive events are shown in red, while CTFR-negative events are shown in blue. A representative plot is shown, with the mean values of size being 88.3 ± 5.9% for CTFR-positive and 11.7 ± 5.9% for CTFR-negative events. For the PBS plot, the mean values are 1.6% for CTFR-positive and 98.4% for CTFR-negative events.

Dynamic Light Scattering (DLS) results: isolation of EVs via device *vs* UC

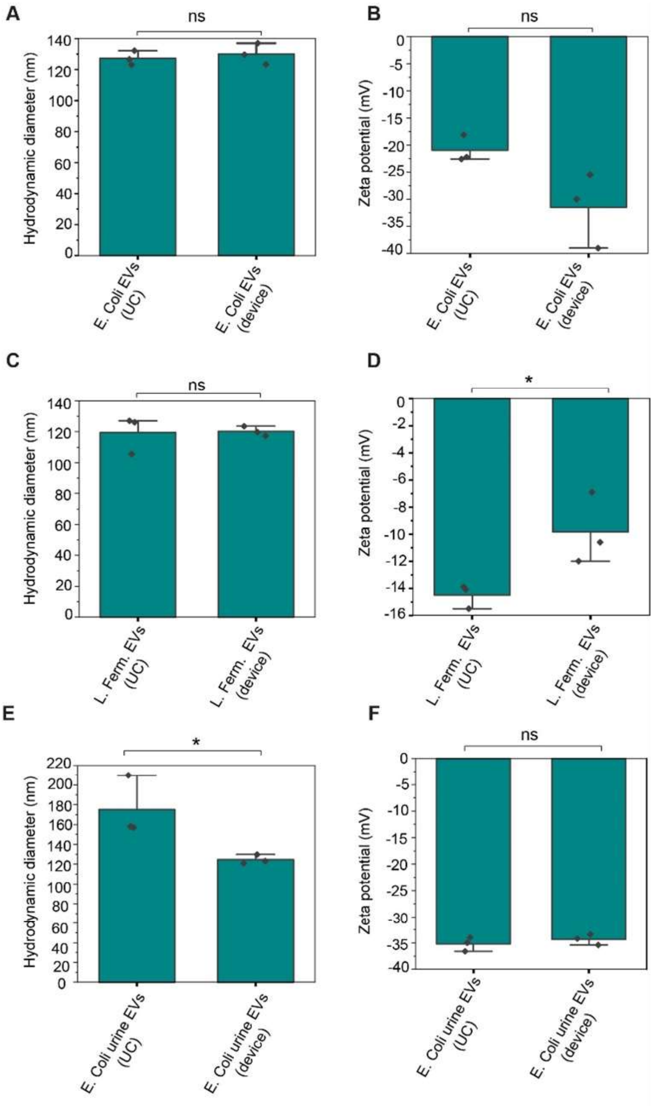

Fig. s5 Top: DLS results obtained from the *E. coli* and *Lb. fermentum* samples collected after isolation with the device compared to UC. (A) The hydrodynamic diameter and (B) the zeta potential values of the EVs after isolation from *E. coli* supernatant by UC (left) and the device (right); (C) The hydrodynamic diameter and (D) the zeta potential values of the EVs after isolation from *Lb. fermentum* supernatant by UC (left) and the device (right); (E) The hydrodynamic diameter and (F) the zeta potential values of the EVs after isolation from *E. coli* contaminated urine sample by UC (left) and the device (right). Statistical significance was calculated by unpaired t-test with a two-tailed distribution and unequal variance. Significance is indicated by an asterisk (*), with ns representing $P > 0.05$. Means ± SD (n=3). Compared to NTA values, the DLS values differ, which is expected due to the distinct measurement principles underlying NTA and DLS techniques. In particular, NTA measures hydrodynamic size based on the Brownian motion of individual particles, while DLS determines size through the scattering of light by particles in suspension.

Table S1: Preparation of BSA standards in a Corning 96-well clear polystyrene microplate for the determination of total protein content of EV samples by Micro BCA Protein Assay

Wells	Water [μL]	Volume (μL) and Source of BSA	Final BSA concentration (μg mL^{-1})
A1-A3	0	300 of 200 μg mL^{-1} BSA	200
B1-B3	150	150 of A1-A3	100
C1-C3	150	150 of B1-B3	50
D1-D3	150	150 of C1-C3	25
E1-E3	150	150 of D1-D3	12.5
F1-F3	150	150 of E1-E3	6.25
G1-G3	150	150 of F1-F3	3.13
H1-H3	150	150 of G1-G3	1.56
A4-A6	150	150 of H1-H3	0.78
B4-B6	150	150 of A4-A6, mix and discard 150	0.39
C4-C6	150	0	0

Table S2: List of antibodies for western blotting

Name	Protein detected	Dilution (v/v)	Manufacturer
Goat anti-mouse IgG/HRP	OmpA	1:2000	Dako Denmark A/S (#P0447)
Goat anti-mouse IgG/HRP	EF-TU	1:2500	Dako Denmark A/S (#P0447)
Goat anti-mouse IgG/HRP	TSG101	1:2000	Dako Denmark A/S (#P0447)
Goat anti-mouse IgG/HRP	CD-9	1:2500	Dako Denmark A/S (#P0447)
Goat anti-rabbit IgG/HRP	ENO1	1:2000	Abcam CB2 0AX, UK (#ab97051)
Goat anti-rabbit IgG/HRP	OmpC	1:1800	Abcam CB2 0AX, UK (#ab97051)
Goat anti-rabbit IgG/HRP	Flagellin	1:1800	Abcam CB2 0AX, UK (#ab97051)
Goat anti-rabbit IgG/HRP	S-layer	1:2000	Abcam CB2 0AX, UK (#ab97051)

Table S3: Summary of the mean hydrodynamic diameter (nm) and zeta potential (mV) values. Data are expressed as mean ± SD (n = 3)

	Hydrodynamic diameter (nm)	Zeta potential (mV)
E. coli EVs (UC)	127.3 ± 4.6	-20.9 ± 2.5
E. coli EVs (device)	130.1 ± 6.9	-31.5 ± 6.9
Lb. ferm. EVs (UC)	119.6 ± 12.2	-14.5 ± 0.8
Lb. ferm. EVs (device)	120.3 ± 3.1	-9.8 ± 2.5
E. coli urine EVs (UC)	175 ± 30.2	-35.2 ± 1.3
E. coli urine EVs (device)	124.6 ± 4.5	-34.3 ± 0.9

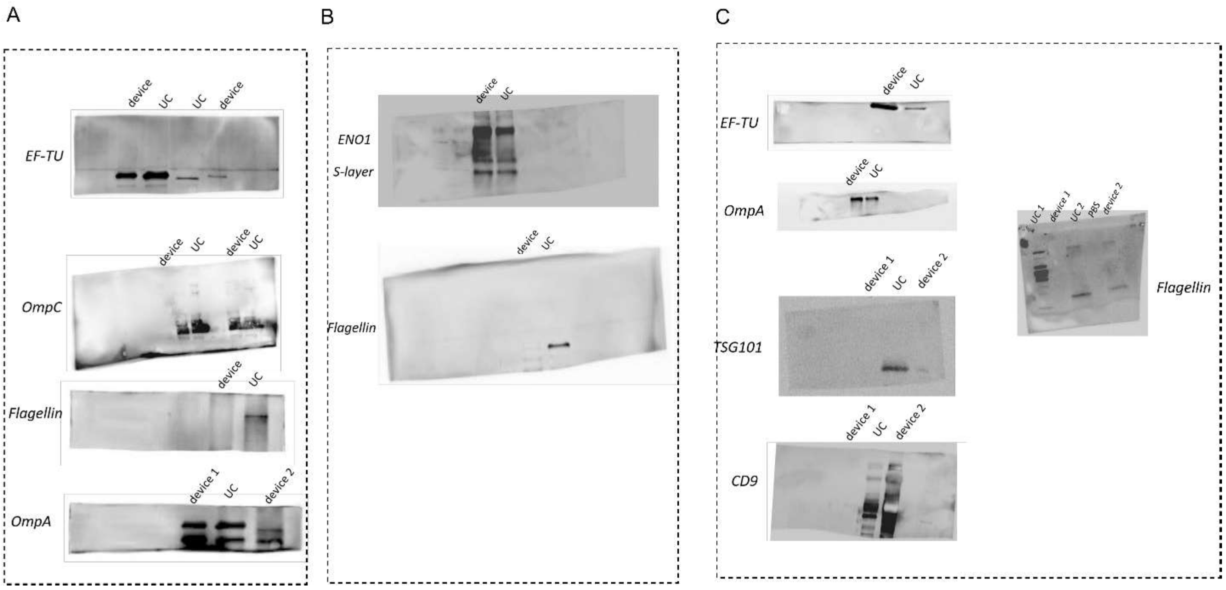

Fig. S6 Original western blots corresponding: (A), (B) and (C) to Figures 2, 3 and 4 in the main manuscript. (A): EF-TU blot: two different sample volumes were run and compared between isolation with device and UC (15 µL in the first two and 5 µL in the other two lanes); OmpC blot: the same samples were run in four different wells; OmpA blot: the two bands could be attributed to the presence of its precursor and mature forms. OmpA is expressed as a precursor protein with a signal peptide, which is cleaved during its translocation to the outer membrane, resulting in a slightly lower molecular weight for the mature form [3]. Device 1 and device 2 differed in the OmpA antibody deposition time on the electrode surface (1 h for device 1 and 20 min for device 2). (B): ENO-1 blot: the presence of multiple bands could be attributed to several factors. ENO-1 may undergo post-translational modifications, resulting in different forms that migrate differently on the gel [4]. The ENO-1 blot was re-incubated with primary rabbit monoclonal anti-bacterial antibody S-layer. Flagellin blot (device): the multiple, yet faint, bands could be due to the protein undergoing post-translational modifications.(C) TSG101 and CD9 blots: devices 1 and 2 differed in the volume of PBS used to wash the electrodes immediately after passing the sample through the channels - 40 mL and 15 mL of PBS in devices 1 and 2, respectively. Flagellin: UC 1 refers to a *Lb. fermentum* EVs sample collected during an optimization experiment, aimed at identifying ENO1. Device 1 and device 2 differed in the PBS volume (15mL for device 1 and 40 mL for

device 2) used to wash the electrodes after introducing the *E. coli* contaminated urine sample. The brightness of the blots was adjusted using the Bio-Rad instrument's Image Transform feature.

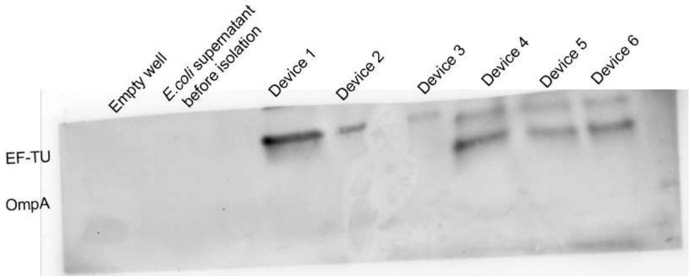

Fig. S7: Original western blot image of OmpA probed from the supernatant of *E. coli* bacterial culture prior to isolation by device or UC. No bands corresponding to OmpA were detected in the unprocessed supernatant. Device 1-6 refer to samples collected during various optimization experiments of the EV isolation process, aimed at identifying EF-TU and OmpA.

References

[1] Gazze SA, Thomas SJ, Parra JG, James DW, Rees P, Durban VM, Corteling R, Gonzalez D, Steven S, Francis LW. High content, quantitative AFM analysis of the scalable biomechanical properties of extracellular vesicles. Nanoscale, 2021; 13: 6129.

[2] Debashish P, Anirban P, Dipanjan M, Saroj S, Manorama G, Suchetan P, Dulal S, Jaydeb C, Samir KP, Tatini R. A Mechanoelastic Glimpse on Hyaluronan-Coated Extracellular Vesicles. J. Phys. Chem. Lett. 2022; 13: 8564–8572.

[3] Nagamine T, Kawasaki W, Iizuka T, Okano K, Matsumoto S, Choudary PV. Functional Characterization of Bacterial Signal Peptide OmpA in a Baculovirus- Mediated Expression System, CSF. 2003: 28; 131-142.

[4] Castaldo C, Vastano V, Siciliano RA, Candela M, Vici M, Muscariello L, Marasco R, Sacco M. Surface displaced alfa-enolase of *Lactobacillus plantarum* is a fibronectin binding protein. Microb. Cell Fact. 2009: 8; 14.
